# The longitudinal associations of material security and belief in God in young Americans

**DOI:** 10.1017/ehs.2025.10035

**Published:** 2026-01-05

**Authors:** Martin Lang, Petr Palíšek, Radim Chvaja

**Affiliations:** 1 LEVYNA: Laboratory for the Experimental Research of Religion, Faculty of Arts, Masaryk University, Brno, Czech Republic; 2 Psychology Research Institute, Faculty of Social Studies, Masaryk University, Brno, Czech Republic; 3 Religion Programme, University of Otago, Dunedin, New Zealand; 4 Faculty of Economics, European Research University, Ostrava, Czech Republic

**Keywords:** cultural evolution, religious systems, secularization, existential security

## Abstract

The prevalence of religious beliefs and practices is puzzling from an evolutionary perspective, but previous research has suggested that religious traditions may provide cooperative benefits and improve well-being. Seemingly in contrast to this claim are worldwide secularization trends in which people disaffiliate from religions and abandon belief in God. Theorists have suggested that diminished pressures on cooperation and well-being no longer motivate individuals to seek religious benefits and pay the associated participation costs. We investigate this claim using the National Study of Youth and Religion dataset, which tracks the development of religiosity among US Christians from adolescence to young adulthood (*n* = 3,370). Using a lagged panel design, we found that material security in Wave 1 (early adolescence) predicts a decrease in belief in God in Wave 4 (young adulthood), although this association is rather small. This result provides some support for the hypothesis that participation in religious traditions is associated with living in an insecure socio-ecology, where religious systems may still confer benefits on their members; yet it is not the only driver of secularization. We conclude with a call for further research using more nuanced measures and larger sample sizes to provide deeper insights into the potentially adaptive nature of cultural systems.

## Graphical abstract

**Figure:**
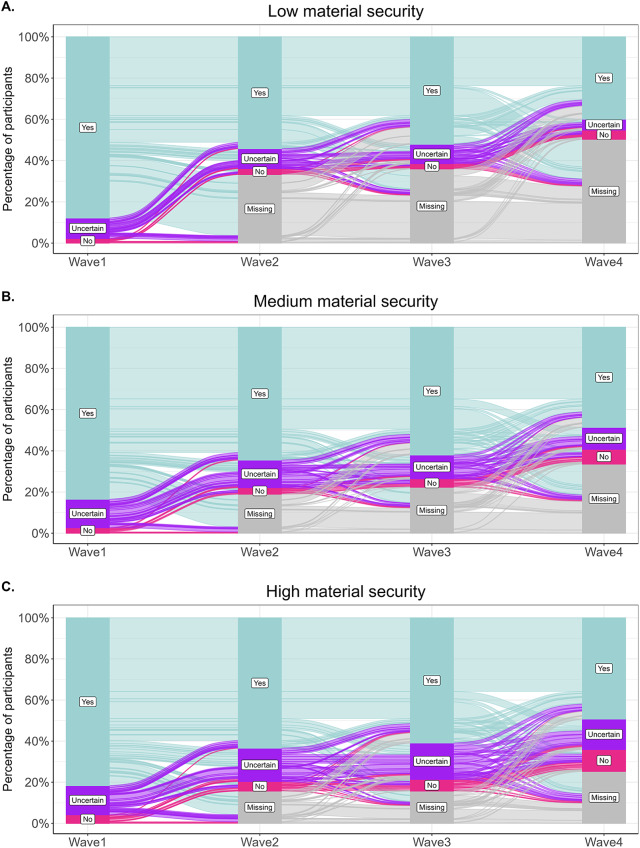

## Social media summary

Material security in adolescence predicts a greater chance of losing belief in God in young adulthood.

## 1. Introduction

The cross-cultural recurrence of religious systems has long puzzled evolutionary scholars, given the apparent costliness of practices and behaviours associated with religious devotion. Previous research has identified two possible benefits of participation in these systems that may offset such costs and help explain their evolutionary stability: (a) positive effects on psychological well-being, health, and survival (hereafter well-being), and (b) positive effects on coordinated and cooperative behaviour (hereafter cooperation).

Current literature generally provides correlational, quasi-experimental, and predictive evidence for the positive associations between religiosity and well-being (Auer et al., 2023; Chatters et al., 2008; Chen & Vanderweele, 2018; Koenig et al., 1999; Powell et al., 2003; Puchalska-Wasyl & Zarzycka, 2019; Sharma & Singh, 2019; Snodgrass et al., 2017; Wallace et al., 2019; Xygalatas et al., 2019; You & Yoo, 2016; see Koenig, 2012 for a review) as well as for the positive relationship between religiosity and cooperation (Ahmed, 2009; Aksoy & Wiertz, 2023; Bulbulia et al., 2024; Chvaja et al., 2025; Galen, 2012; Green & Elliott, 2010; Johnson et al., 2016; Lang et al., 2019; Purzycki et al., 2016; Saroglou et al., 2005; Shariff & Rhemtulla, 2012; Sosis & Handwerker, 2011; Stavrova & Siegers, 2014; Van Cappellen et al., 2016; Xygalatas et al., 2013; see Kelly et al., 2024; Shariff, 2015; Tsang et al., 2021 for a review).

Controlled experimental manipulations further hint at the possible causal nature of this relationship. For example, reminding participants of omnipotent supernatural agents may act as an anxiety buffer (Good et al., 2013; Inzlicht & Tullett, 2010), and religious rituals may help decrease anxiety (Karl & Fischer, 2018; Lang et al., 2020, 2022; Sosis & Handwerker, 2011). Similarly, reminders of supernatural beliefs have been shown to prompt cooperative behaviour (Clobert et al., 2015; DeBono et al., 2017; Pasek et al., 2023; Preston & Ritter, 2013; Shariff et al., 2016; White et al., 2019), and similar results were obtained when using the reminders of religious rituals in the laboratory (Aveyard, 2014; Lang et al., 2016; Nichols et al., 2020) and in real religious settings (Rand et al., 2013; Xygalatas, 2013).

This preliminary support needs to be interpreted with caution, however, since recent experiments, meta-analyses, and reviews suggest that religious concepts and manipulations do not uniformly promote cooperation (Ge et al., 2019; Gomes & McCullough, 2015; Jackson & Gray, 2019; Major-Smith, 2023; van Elk et al., 2015). Instead, evidence indicates that the cooperative effects of religiosity are largely parochial, fostering cooperation primarily among co-religionists (Isler et al., 2021). Likewise, the positive association between religiosity and well-being appears to be conditional and often weak (Prati, 2025). This is particularly evident in contexts with anti-religious norms (Stavrova et al., 2013), or when individuals rely on negative religious coping strategies to confront adversity (Pirutinsky, 2024).

Yet further indirect support for the purported benefits of religious systems comes from observations that religiosity intensifies following threats to well-being and cooperation. For example, people in geographical areas with a higher probability of natural disasters or who have recently experienced such disasters display higher religiosity and religious affiliation (Sibley & Bulbulia, 2012; Sinding Bentzen, 2019) and the same is true for those more exposed to the negative consequences of the recent Covid-19 pandemic (Bentzen, 2021; Chvaja et al., 2024; Kanol & Michalowski, 2023). Wars are another systemic shock that has been shown to increase religiosity (Keinan, 1994; Shai, 2022) even several years after the conflict (Henrich et al., 2019) and also in neighbouring countries (Chvaja & Lang, 2024). Furthermore, a longitudinal study from Indonesia showed that participants who were more affected by inflation were more likely to engage in communal Koran study and to send their children to Islamic schools (Chen, 2010), and similar associations were found during US agricultural and housing crises (Orman, 2019).

Experimental manipulations mirroring the naturally occurring pressures on well-being and cooperation further suggest religion may play a role in responding to the threats of personal well-being and group cooperation. For instance, increasing insecurity leads to higher self-reported scores on belief in omnipotent deities (Kay et al., 2008; Laurin et al., 2008) and similar findings were reported for ritual behaviour (Karl & Fischer, 2018; Lang et al., 2015) and superstitious practices (Keinan, 2002). In terms of cooperation, experimental manipulations of trust breaches increase the probability of reporting that greed angers gods (Purzycki et al., 2020) and of joining religious groups securing cooperation (Lang & Chvaja, 2024). When considering hypothetical future scenarios, participants reported that it would be more important for people to believe in a punitive god in a scenario including wars (Caluori et al., 2020). Taken together, despite the preliminary nature of some findings and the presence of mixed results, the available evidence converges on a picture in which religious traditions support their members in adverse times by fostering cooperation, enhancing perceived control, and reducing anxiety, thereby promoting well-being. These benefits may help explain, at least in part, the widespread prevalence of such cultural systems.

### 1.1. Secularization

In a seemingly stark contrast to the evidence supporting the benefits provided by participating in religious systems, many countries worldwide have exhibited gradually decreasing rates of religiosity during the 20th century, a phenomenon labelled as ‘secularization’ (Berger, 1967). The occurrence and intensity of secularization have been debated for decades now (for an overview, see Bruce & Voas, 2023; Stark, 1999; Stolz & Voas, 2023), with the revival of religiosity in post-Soviet countries during the 1990s and the persistence of religiosity in the United States often given as counterexamples to the secularization thesis (Stark, 1999; for more nuanced support of these counterexamples, see Northmore-Ball & Evans, 2016; Schnabel & Bock, 2017; compare Stolz et al., 2023). Nevertheless, longitudinal evidence from the multiple waves of the World Values Survey suggests that secularization occurs worldwide and has recently intensified (Inglehart, 2020). Why would people leave religious traditions that, according to research cited above, supply them with essential benefits?

One potential explanation congruent with the proposed role of religious systems that react to adversity by regulating cooperation and well-being-related practices posits that secularization is driven by the diminution of the pressures these systems respond to. Developed as the existential insecurity theory by Norris and Inglehart (2004), this account proposes that economic development has increasingly brought political, financial, and personal stability that made the world a much safer place, thereby diminishing the role of religion in assuaging anxieties and fears arising from insecure and uncontrollable environments.

In support of this proposition, Norris and Inglehart (2004) showed that countries with greater socio-economic inequality are more religious, while countries with a higher human development index are less religious. Over the last two decades, this thesis has received much empirical support, with studies showing that the Gini index tracking income inequality is positively associated with religiosity both between and within countries (Barber, 2011; Kusano & Jami, 2022; Pew Research Center, 2018; Ruiter & Van Tubergen, 2009; compare Höllinger & Muckenhuber, 2019), while higher GDP, income, and social welfare are negatively related to religiosity (Barber, 2011; Dhima & Golder, 2021; Gill & Lundsgaarde, 2004; Immerzeel & Van Tubergen, 2013; Ruiter & Van Tubergen, 2009; but see Te Grotenhuis et al., 2015). Finally, evidence from 15 small-scale societies further shows that perceived insecurity over future food availability is positively associated with commitment to moralizing deities (Baimel et al., 2022; cf. Purzycki & Bendixen, 2024).

One potential explanation of these results is that material security brought by economic development may ameliorate the pressures on well-being and cooperation and, in return, decrease the need for participation in religious traditions. Indeed, household income is associated with trustworthy cooperative networks as well as access to quality healthcare in the United States (Brandt et al., 2015; Giordano et al., 2019; Kanter et al., 2020). However, most evidence supporting the dynamic process between material security and religiosity comes from cross-sectional data. That is, we lack evidence on the level of an individual that material and physical security precede decline in religiosity.

Furthermore, the process of disaffiliation is most intensive during adolescence and young adulthood, when individual identities are forged based on individual experiences in the given environment and through interaction with vertical and horizontal cultural transmission (Alcorta & Sosis, 2005; Arnett, 2000; King et al., 2021). Such identities formed in adolescence may affect a person’s political and religious views later in life, calling for greater focus on longitudinal data capturing this transition. That is, if security indeed positively affects religious disaffiliation, it should be most evident in the turbulent period of adolescence.

### 1.2. Adolescence

Norris and Inglehart (2004, p. 18) proposed that secularization is driven by the socio-environmental context of ontogeny, a sentiment echoed in Inglehart’s (2020) later work, where he lamented the lack of appropriate data to test this proposition. Indeed, the transition from adolescence to young adulthood is often accompanied by changes in religious commitment in Western countries (Chan et al., 2015), manifesting mainly as a decreased frequency of attending religious services (Lopez et al., 2011; Regnerus & Uecker, 2006).

A key factor in adolescents’ religiosity and its purported change later in life is the nature of socialization (or lack thereof) into religious groups, most often facilitated by their close family (Crockett & Voas, 2006; Mikoski & Olson, 2021; Storm & Voas, 2012). For example, recalled parents’ attendance of religious services during participants’ childhood is a statistically significant predictor of participants’ contemporary religious commitment (Willard & Cingl, 2017). However, note that while the fidelity of this transmission process is crucial for passing on religiosity to the next generation (Legare, 2019; J. Smith, 2021), the existential insecurity theory predicts that it is the material security surrounding the growing adolescent that should affect the development of religiosity (Norris & Inglehart, 2004), pushing children to resist their parents’ teachings (Stolz, 2020). In support, a retrospective study by Immerzeel and Van Tubergen (2013) showed that having unemployed parents when 14 predicts higher adult levels of religiosity across 26 European countries, and the same is true for people who grew up during a violent conflict, although this association is rather small (0.2 higher score on a 0–10 religiosity scale).

Several studies have used available longitudinal data to test factors affecting the purported decline of religiosity during the transition from adolescence to young adulthood. While some suggest that obtaining an undergraduate degree is associated with a sudden drop in religiosity among US participants (Schwadel, 2016), others observed that the decrease in religiosity was most pronounced among young adults who did not pursue higher education (Uecker et al., 2007). Uecker et al. also found that getting married tempers the decline in religiosity while cohabitation accelerates it, and the same is true for illicit/non-normative behaviour, suggesting that engaging in behaviours incongruent with religious norms leads to abandonment thereof. Looking at personality characteristics predicting religious change, Mccullough et al. (2005) found that emotional instability during adolescence predicted the retention of religiosity, that is, prolonged the effects of religious socialization. While insightful, these previous longitudinal studies did not focus on how experience during childhood and adolescence might predict religiosity later in life (e.g., in participants’ 20s).

### 1.3. Current study

To fill this gap and test whether material security during adolescence predicts a later decline in religiosity, we report a preregistered analysis (https://osf.io/s6nmg/) of the National Study of Youth and Religion (NSYR) longitudinal dataset (C. Smith, 2021; C. Smith & Denton, 2003) that contains four data-collection waves among US adolescents and young adults (from ages 13–17 in Wave 1 to 23–28 in Wave 4). We assess whether material security during adolescence (operationalized as parents’ income) would be predictively associated with self-reported belief in God in the later data-collection waves. We rely on the estimation of the predictive associations (sometime referred to as Granger causality) in this study since this approach allows us to test a more modest claim – namely, about the temporal precedence and predictive utility of the exposure variable for the outcome variable in time-series data. While this temporal precedence is a necessary condition for a causal relationship between material security and religious belief, we do not directly estimate the causal relationship – an approach that would require a hypothetical intervention on the exposure variable (e.g., Bulbulia et al., 2024).

Regarding the focal variables operationalizing the predictions from the above-reviewed theories, we use parents’ income as a proxy for material security. While income does not represent the broad scale of insecurities listed by Norris and Inglehart (2004), economic measures such as GDP are often used as predictors by the proponents of existential insecurity theory, and Norris and Inglehart themselves reported that household income is negatively related to religiosity. Similarly, we focus on belief in God as the defining aspect of commitment to religious systems. While multiple aspects of religious systems may promote the benefits described above (Lang & Kundt, 2020), belief in God is often the defining aspect of religious systems, differentiating these systems from other cultural systems that may provide similar benefits (Atran & Norenzayan, 2004; Boyer, 2001; Lawson & McCauley, 1990; Norenzayan, 2013). Moreover, losing faith in God is the crucial aspect of secularization that differentiates secularization from other social processes in which religion may lose power or shift into different social systems (as described in Stark, 1999, pp. 251–252). Thus, if we aim to study the demise of religious systems, we need to study the demise of their core aspect – at least in the major religious traditions present in the United States – namely, belief in God.

## 2. Methods

### 2.1. Context of the current study

The United States has long been considered an exception from the secularization process taking place in other Western countries (Bruce & Voas, 2023; Stolz, 2020). Yet, since the 1990s, secularization has also accelerated in the United States, manifesting in a decreasing percentage of people affiliated with religious organizations. While from the 1970s to the early 1990s around 90% of the US population identified as Christian, this number fell to 63% in 2021 (Pew Research Center, 2022). During the same period, affiliation with other religious traditions hovered around 7%, while the proportion of the religiously unaffiliated rose from 5% to 29%. Similarly, the percentage of respondents who never attended church increased from 13% in 1990 to 26% in 2014 (Voas & Chaves, 2016). Although some argue that this decline is driven primarily by disaffiliation among the moderately religious, with the proportion of highly committed believers remaining stable (Schnabel & Bock, 2017), others contend that the United States follow the secularization trajectory of other Western countries, only somewhat delayed (Brauer, 2018; Crockett & Voas, 2006; Stolz, 2020).

Some researchers have also argued that these changes do not necessarily reflect an increase in non-theism but rather a decline in religious affiliation, coining the term *spiritual but not religious* (Fuller, 2001; Heelas et al., 2004; Willard & Norenzayan, 2017). However, the percentage of participants reporting that they do not believe in God rose from 4% in 1990 to about 23% in 2017. Similarly, while 65% of respondents aged 65 and older reported belief in God without a doubt in 2018, only 39% of respondents aged 18–34 did so (Inglehart, 2020). Thus, although some of the disaffiliation trends in the United States may be explained by transformation into alternative forms of spirituality, the secularization process is nevertheless underway, including our main outcome variable – belief in God. Moreover, the data used in the current study were collected between 2002 and 2013, directly overlapping with the major wave of secularization described above.

One caveat to our approach is the financial crisis the United States experienced in 2007–2008. Although the timing of this crisis falls outside the window of adolescence for our participants and would not affect material security in Wave 1, it likely reshaped material security unevenly across the sample afterwards. Nevertheless, we assume that the crisis primarily reinforced existing disparities in material security, as low-income households were more significantly affected (especially in the long term) than high-income households (Fligstein & Rucks-Ahidiana, 2016).

### 2.2. National study of youth and religion data set

The first wave of data collection was conducted between 2002 and 2003 with 3,370 children aged 13–17 and their primary caregivers (referred to as parents henceforth for simplicity). The second wave of data collection took place in 2005, and the third between 2007 and 2008. Approximately 78% of the initial youth survey participants were successfully re-interviewed in Wave 2 (then aged 16–20), and 77% were re-interviewed in Wave 3 (aged 18–23). Finally, 67% of the original youth respondents participated in Wave 4, when they were aged 23–28 (C. Smith & Denton, 2003). See Supplementary Material, Section 1 for an overview of the previous analyses of the NSYR dataset on related topics.

### 2.3. Selected variables

**Figure 1.**
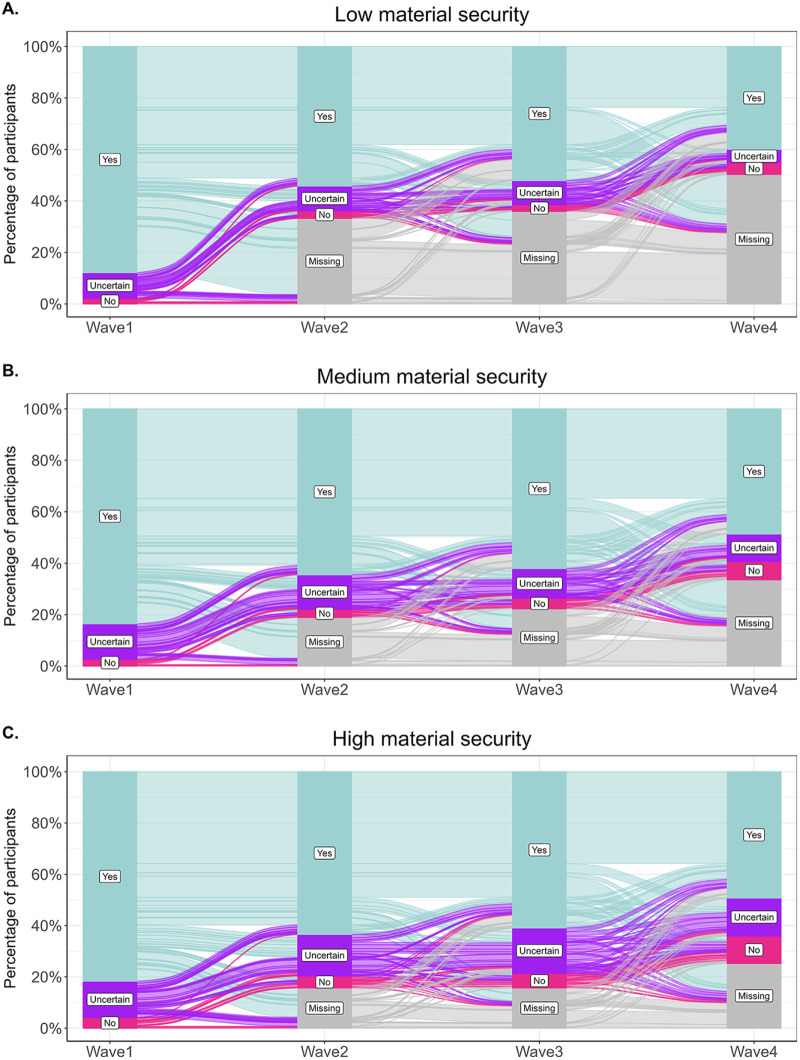
An alluvial plot displaying raw trends in belief in God across the four waves of data collection.

The core relationship modelled in our study is between material security (Wave 1) and belief in God (Waves 2–4). Material security was operationalized as parent-reported annual household income (before taxes) in Wave 1, coded on an 11-point scale corresponding to $10,000-wide income bands ranging from less than $10,000 to more than $100,000. Belief in God was measured with a single item asking whether the participant believes in God, with response options ‘No’, ‘Don’t know/unsure’, and ‘Yes’. We treated this variable as ordinal, with the ‘Unsure’ category positioned between ‘Yes’ and ‘No’ to represent participants in transition (as illustrated in Fig. 1).

We divided the plot according to material security in early adolescence, our main predictor variable (which was categorized from an ordinal variable for the purpose of this plot, see Methods). The *Y*-axis displays the percentage of people in each material security category answering ‘Yes’, ‘Uncertain’, ‘No’ or was missing on the question of belief in God. The number of people in each category: Low = 1,186, Medium = 1,060, High = 917.

**Figure 2.**
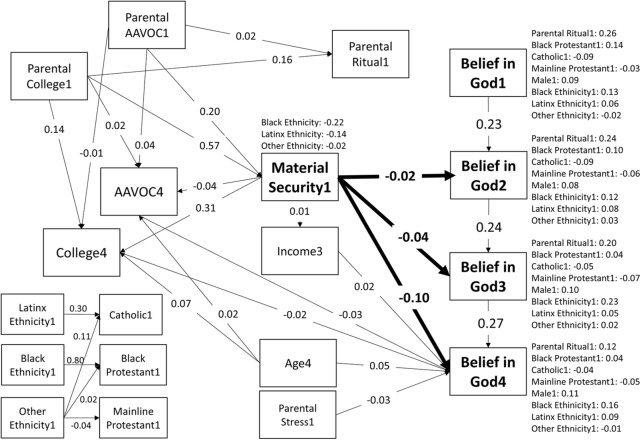
Results of the main hypothesis test with controls.

Thinking about possible confounds of the predictive associations between material security (Wave 1) and belief in God (Waves 2–4), we built a Directed Acyclic Graph (DAG; McElreath, 2016; Pearl, 2009) using variables available in the NSYR dataset. The full DAG was tested as the controls model, as shown in Fig. 2. In the DAG, we identified parents’ education and parents’ ethnicity as potential confounders. Both variables can influence parents’ material security as well as the type of religious upbringing participants received. We operationalized parents’ education as the highest level of educational attainment reported for either parent in Wave 1. The resulting categories were ‘both parents college’, ‘one parent college’, ‘both parents high school or vocational school’, ‘one parent high school or vocational school’, and ‘neither parent high school nor vocational school’. Similarly, we identified ethnicity as a potential confounder, since material security differs by ethnicity in the United States, and the same is true for patterns of religiosity (Barber, 2015). Ethnicity was self-reported by parents in Wave 1. Where ethnicity was incongruent between parents, we used the ethnicity congruent with the one self-reported by the participant in Wave 1. Categories for ethnicity were ‘White/Caucasian’, ‘Black/African American’, ‘Hispanic/Latinx’, ‘Other’.

The main estimated paths are in bold, other paths served either as controlling for potential confounding or explaining variability in belief in God. Numbers on arrows denote standardized beta estimates (note: Table 3 reports unstandardized estimates). Numbers next to variable names indicate wave in which the variable was collected. We used ethnicity as predictors of material security and religious affiliation and both as predictors of belief in God. These relationships are stated verbally (without arrows) to limit cluttering of the main tested relationships. Correlations are omitted. Reference categories for categorical predictors: Gender – Women; Ethnicity: White; Religious tradition: Conservative Protestantism; Education: no/high school. AAVOC = Associate degree or vocational school graduate.

Our model further includes variables that do not necessarily confound the focal relationship but may modify the detected impact of the predictor variables on the outcome (Digitale et al., 2022). Specifically, we include participants’ gender, as women often report higher levels of commitment to deities such as the Christian God (Vardy et al., 2022). Likewise, the frequency of reporting belief in God may differ across religious denominations, so we included this variable in the model as well, although it could be argued that, since our sample is predominantly Christian, denominational differences should not substantially affect the overall association. The denominations represented were ‘Catholic’, ‘Mainline Protestant’, ‘Black Protestant’, and ‘Conservative Protestant’. Similarly, because religiosity often fluctuates with age, especially during young adulthood (Major-Smith, 2023), we included age as an adjusting variable.

We also adjust our model for participants’ highest level of attained education since this relationship has been reported previously (Schwadel, 2016) and often constitutes an alternative explanation to existential insecurity theory (e.g., Berger, 1967; Wilson, 1966). In short, the modernization theory proposes that rationalization and mechanization of the world, symptomatic of the modernization process in industrialized societies, ‘disenchant’ the world and leave little space for religious beliefs that are logically or rationally indefensible (Bruce, 2011). These effects are further amplified by increasingly accessible education that trains people in critical thinking, which is in many respects antithetical to religious beliefs (Bruce, 2011). Indeed, years of education are positively correlated with secularization (Schwadel, 2015, 2016; but see Uecker et al., 2007) and this effect is independent of the effect of insecurity (Pollack, 2008). Thus, if educational achievement were to mediate the effect of material security on later belief in God, we would not consider this pathway to support the existential insecurity theory. Education was operationalized as participants’ highest level of formal attainment in Wave 4 (ranging from ‘no degree’ or ‘high school’ to ‘vocational school’ and ‘graduate’), assuming that the effects of completing a degree preceded its reporting in Wave 4. Note that we deviated from our preregistration by collapsing the ‘no degree’ and ‘high school’ categories, as the ‘no degree’ option was represented by only 3.8% of the sample.

Similarly, other variables in the model were included to eliminate potentially mediating pathways not proposed by existential insecurity theory. These included parental stress in Wave 1 and participants’ income in Wave 3, which may capture effects of material security but not during the hypothesized period of adolescence. Furthermore, as explained in the introduction, we added parental ritual frequency in Wave 1, which may affect the intergenerational transmission of religiosity and thus constitutes an alternative explanation. While we preregistered only the path between parental ritual frequency and belief in God in Wave 4, following peer review we added paths from parental ritual frequency to belief in God in Waves 1–3, a step consistent with our overall modelling approach (analogous to how material security in Wave 1 predicts belief in God across Waves 1–4). See the R code in the OSF registry associated with this project for further details on how these additional variables were modelled.

Note that we also preregistered a more complex model that included mediation pathways between self-reported health (as a proxy for the pressure on well-being), self-reported trust in others (as a proxy for the pressure on cooperation), and prayer frequency and collective ritual participation frequency (as potential mediators between pressures and belief). While this complex model aimed to differentiate between the presence of specific pressures and their association with belief in God as well as whether a decrease in ritual behaviour might precede a decrease in belief, these mediating pathways were assessed only by single-item variables, and the model exhibited poor fit to the data. Furthermore, after peer review, the reviewers pointed out that our causal assumptions were insufficient to properly test these mediating pathways. We report this model in the Supplementary Material, Section 2, but note that this model is highly exploratory and future work should test the proposed pathways with more precise latent variables and additional causal assumptions.

### 2.4. Analysis

The analysis was conducted in R (v. 4.5.0), using the *lavaan* (v. 0.6-19; Rosseel, 2012), MASS (v. 7.3-65; Venables & Ripley, 2002), semTools (v. 0.5-7; Jorgensen et al., 2022), lavaan.mi (v. 0.1-0; Jorgensen et al., 2022), and *restriktor* (v. 0.6-10; Vanbrabant & Kuiper, 2024) packages. As preregistered, for all the reported analyses, we excluded participants who reported no belief in God in Wave 1 because the assumed process of socialization into religious belief either did not take place or was not successful; hence, we cannot measure how material security modifies the effects of religious socialization in these participants (as predicted by Norris & Inglehart, 2004). We also excluded non-Christian participants since their low numbers would not allow us to reliably establish the proposed effect in other religious traditions (we assume some of the proposed trajectories may be tradition-specific). For participants who were unaffiliated or indeterminate but believed in God in Wave 1 and at least one of their parents was Christian, we retained them in the analyses, assuming that supernatural belief would likely be affected by Christianity.

Regarding our analytical strategy, we applied the principles of a General Cross-Lagged Panel Model (GCLPM; Zyphur et al., 2020) to assess predictive associations and to differentiate between short- and long-term effects of material security on disbelief in God, as well as to test whether first-wave effects are more prominent than later-wave effects. However, our main predictor variable – material security during adolescence – was assessed only in Wave 1. Therefore, we built a lagged panel model (not cross-lagged) that allowed us to estimate: (1) an intercept for each variable of interest collected across multiple waves, accounting for general trends in the data; (2) unit effects of belief in God to account for varying intercepts between participants, with factor loadings fixed to 1 (except at the first occasion) to ensure stability across waves; (3) autoregressive terms for variables measured across more than one wave; and, most importantly, (4) higher-order lagged terms linking material security in Wave 1 to belief in God in Waves 2–4. These higher-order lagged terms allowed us to quantify and compare the temporal dynamics of material security’s effect on belief in God. Furthermore, our preregistered model conceptualized belief in God as an ordinal variable with theta parameterization (Jak & Jorgensen, 2023) with thresholds constrained to be equal across waves.

To formally test our hypothesis, we used informative hypothesis testing using the Generalized Order-Restricted Information Criterion Approximation (GORICA; Kuiper & Hoijtink, 2013; Kuiper et al., 2011; Vanbrabant et al., 2020). GORICA is an Akaike-type information criterion for an evaluation of order-restricted hypotheses, and it has been applied to multiple statistical models, including the random-intercept cross-lagged panel model, to evaluate the empirical fit of a hypothesis penalized by its complexity (Sukpan & Kuiper, 2023). The resulting inference is based on assessing GORICA weights, which, like Akaike weights, quantify the relative support for a given hypothesis compared to the rest of possible hypotheses. For example, a GORICA weight of 0.80 compared to the one of 0.20 means the first hypothesis is four times more likely than the second one. GORICA weights can be interpreted using benchmarks, which construct credible intervals around the weights’ estimates (Vanbrabant & Kuiper, 2024). To facilitate a more accurate interpretation of GORICA weights, we use the degree of overlap of the intervals for the null and the observed associations when assigning verbal labels (e.g., ‘overwhelming evidence’) to our results. We also provide traditional model comparison using fit indices as a sensitivity check in the Supplementary Material.

We first fitted a baseline model assessing the relationship between material security in Wave 1 and belief in God in Waves 2–4. Next, we added all planned control variables and their interrelationships. For each of these models, we freely estimated the proposed paths and subsequently compared the relative support for each hypothesis using GORICA. As a sensitivity check, we also built a simple linear model assessing only the association between material security and belief in God in Wave 4 (ignoring earlier measures of belief in God), and then added control variables to this model. We further explored how different analytical choices might have influenced our conclusions by estimating models with pairwise missing-data handling as well as models that treated the manifest variables as continuous using maximum likelihood (ML) estimation. See Section S4 of the Supplementary Material for the results of these sensitivity checks (which generally support the reported findings) and Section S5 for an overview of deviations from the preregistration.

### 2.5. Missing data handling

In our preregistration, we planned to listwise exclude participants with missing data on focal variables for each hypothesis test. If listwise deletion were to exceed 10% of the sample, we planned to use multivariate imputation techniques. Ultimately, because of the substantial amount of missing data (see Table 1 for details), we performed multivariate imputation (70 imputed datasets, 20 maximum iterations) using chained equations with the *mice* R package (Van Buuren & Groothuis-Oudshoorn, 2011). Ordinal variables were imputed using proportional odds logistic regression, continuous variables using predictive mean matching, and binary variables using logistic regression. In this way, we imputed all variables included in our models. Although our analyses below do not include participants who did not fulfil exclusion criteria (see above), their data were used for imputation purposes. The imputation procedure completed without any logged errors and passed visual convergence checks. Three participants were removed from the analyses based on the imputed datasets because the procedure did not impute a value for material security, likely due to predictor sparsity. See Section 5 of the Supplementary Material for additional checks of imputation assumptions.

**Table 1.** Belief in God across waves

Wave	No	Uncertain	Yes	Missing
1	0	313 (11%)	2519 (89%)	0
2	63 (3%)	319 (15%)	1778 (82%)	672 (24%)
3	83 (4%)	308 (15%)	1714 (81%)	727 (26%)
4	152 (9%)	248 (14%)	1342 (77%)	1090 (38%)

*Note:* Relative frequencies in columns ‘No’, ‘Uncertain’, and ‘Yes’ are computed for non-missing data only. The ‘Missing’ column shows relative and absolute frequencies of missing values in each wave.

All reported estimates are pooled based on Rubin’s rules over the imputed datasets using the *semTools* function *runMI* (Jorgensen et al., 2022); GORICA estimates are based on these pooled results, extracted using the *lavaan.mi* package (Jorgensen et al., 2022). Robust fit indices were obtained by scaling the pooled values by the average scaling factor (see *semTools* documentation for details; Jorgensen et al., 2022).

## 3. Results

### 3.1. Descriptive results

Following data cleaning procedures, our final sample consisted of *N* = 2,832 unique participants, with 50% women and 50% men. Most (65%) participants reported identifying as White but there were also sizable groups of Black (19%) and Latin (11%) participants. The majority of participants reported affiliating either with Protestantism (63%) or Catholicism (29%). A substantial portion of our sample (41%) were college educated. Median reported parental income was between 40,000 USD and 50,000 USD (5th category out of 11) with inter-quartile range of 4, that is, from categories 30,000–40,000 USD (4) to 90,000–100,000 USD (8). Table 1 presents descriptive statistics for the belief in God variable across the four waves. See also Fig. 1 for visualization of the focal assessed relationship.

### 3.2. The association of material security on belief in god

The baseline model containing material security in Wave 1 and belief in God across Waves 2–4 without any controls (except the autoregressive controls of belief in God across Waves 1–3) supported the hypothesized association. The pooled baseline model, having a small number of degrees of freedom, exhibited a very good fit: χ^2^(8) = 22.39, *p* = 0.004, *D* = 0.72, RMSEA = 0.025, _90%_CI [0.013; 0.038], SRMR = 0.014, TLI = 0.988 (note that although the fit indices are based on the scaled version of χ^2^, they do not account for the ordinal nature of the data; Jak & Jorgensen, 2023). The combined unstandardized association between material security in Wave 1 and belief in God in Wave 4 (i.e., this association includes the association between material security and belief in God from previous waves due to is propagation via auto-regressive paths) was *b* = −0.10 _95%_CI [−0.13; −0.06], *p* < 0.001). According to GORICA, this hypothesis is 9,854 times more likely than the complement in this baseline model – recall that the complement contains all other possible orderings of the effects apart from the effects hypothesized (Vanbrabant et al., 2020). In terms of raw data, this result convenes with the observation that 5% of participants in low material security (as categorized in Fig. 1) who believed in God in Wave 1 said that they do not believe in Wave 4, while in the high material security group, it was 11% of the sample. See Table 2 for the focal model estimates and Table S3 in the Supplementary Material for all model parameters.

**Table 2.** Pooled results of the baseline model

		*b*	SE	z	*p*
*Auto-regressive paths*					
Belief in God1 -> Belief in God 2		0.34	0.06	6.06	<0.001
Belief in God2 -> Belief in God 3		0.32	0.04	8.46	<0.001
Belief in God3 -> Belief in God 4		0.33	0.04	9.11	<0.001
*Lagged paths*					
Material Security1 -> Belief in God2		−0.03	0.02	−1.96	0.049
Material Security1 -> Belief in God3		−0.04	0.02	−2.77	0.006
Material Security1 -> Belief in God4		−0.08	0.02	−4.86	<0.001

*Note.* *b* = unstandardized regression coefficient; SE = standard error of *b; z* = *z*-statistic value; *p* = *p*-value for the *z*-statistic. Numbers next to variables denote data-collection waves.

Next, we present the model with controls, testing the predictive association posited in our main hypothesis. The model is based on a DAG designed to capture the temporal precedence of material security while controlling for potential confounds to the detected predictive associations. See Fig. 2 for the full list of potential confounds and their modelled relationships.

The global fit index values for the model with controls are borderline, but still acceptable given the lack of concerning residual correlations: χ^2^(97) = 635.13, *p* < 0.001, *D* = 1.13, RMSEA = 0.044 _90%_CI [0.041; 0.048], SRMR = 0.032, TLI = 0.639. Nonetheless, this model with controls revealed a worse fit to data compared to the baseline model, especially in terms of TLI. However, the low TLI value reflects the lack of correlations between the model variables, which makes the null model fit extremely well: in our case RMSEA_null_ = 0.074. Since using the traditional fit index cut-offs is no longer considered best practice and local fit assessment is preferred instead (Kline, 2024), we also inspected the model residuals, averaged over the imputed datasets. We found positive residuals between some levels of categorical variables (e.g., levels of religious tradition and education) and a large negative residual for the association between the dummy-coded ‘College’ variable and ‘Income’ in Wave 3 (approximately –0.20). Both issues were remedied by freely estimating these correlations. Although this procedure can be considered somewhat data-driven, it is important to note that all versions of the model produced largely similar estimates of the hypothesized association, suggesting that the overall model fit was acceptable. We therefore base our main inferences on this final model, which achieved the best fit and for which residual matrices were cross-checked.

The summed unstandardized size of the main hypothesized relationship was found to be *b* = −0.07 (_95%_CI [−0.11; −0.03], *p* < 0.001), which is 16.90 times likelier than the complement according to the GORICA statistic. This ratio is higher than the 95th percentile of the population ratios when assuming no association (3.63). While the effect size is a third lower compared to the baseline model and the GORICA support dropped massively, this result still represents substantial support for the given hypothesis. That is, since we added important demographic controls, it is reasonable to expect that these controls would explain major portion of variance in the data, as could be also seen in the decreased estimates for the auto-regressive paths (compare these paths between Tables 2 and 3). See Table 3 and Fig. 2 (Table S4 for other parameters of this statistical model).

**Table 3.** Pooled results of the model with controls

		*b*	SE	*z*	*p*
*Auto-regressive paths*					
Belief in God1 -> Belief in God 2		0.31	0.07	4.26	<0.001
Belief in God2 -> Belief in God 3		0.26	0.05	5.60	<0.001
Belief in God3 -> Belief in God 4		0.27	0.04	6.07	<0.001
*Lagged paths*					
Material Security1 -> Belief in God2		−0.01	0.02	−0.60	0.550
Material Security1 -> Belief in God3		−0.03	0.02	−1.67	0.094
Material Security1 -> Belief in God4		−0.06	0.02	−3.40	<0.001

*Note.* *b* = unstandardized regression coefficient; SE = standard error of *b; z* = *z*-statistic value; *p* = *p*-value for the *z*-statistic. Numbers next to variables denote data-collection waves. These associations were adjusted for variables mapping participants’ religious denomination, ethnicity, education, parental education, parental ritual, income in Wave 3 (scaled), age (scaled), and the relationship between these variables (see Supplementary R script for the full model specification).

### 3.3. Sensitivity analysis

To account for the various ways our analysis could plausibly be specified, we fit the same models as above in four additional ways: (1) using a regression model with a clog-log link with only belief in God in Wave 4 (as requested by a reviewer); (2) as an structural equation model ignoring the ordinal nature of the outcome variable, estimated using maximum likelihood with robust standard errors; (3) the same as (2) but without multiple imputation, instead relying on full information maximum likelihood; and (4) without multiple imputation, treating missing data with the pairwise deletion approach. The results of these sensitivity checks, reported in Section 4 of the Supplementary Material, showed that the main hypothesis is strongly supported in most specifications. However, the association between material security and belief in God is attenuated when the ordinal nature of the outcome variable is disregarded (i.e., in the maximum-likelihood models).

## 4. Discussion

This paper sought to investigate the predictive association (often described as Granger causality) between early adolescent material security and disbelief in God in young adulthood, testing whether such security during the maturation process may be associated with later support for cultural systems that manage pressures on cooperation and well-being, namely religion. Whereas previous theories have suggested that experiences during adolescence should predict religiosity in adulthood (Inglehart, 2020; Norris & Inglehart, 2004), this claim has largely remained untested due to the absence of longitudinal data tracking the individual-level development of beliefs from adolescence. Moreover, the cross-sectional nature of previously used data did not allow researchers to test the temporal precedence of security relative to losing belief in God. We harnessed the longitudinal design of the NSYR dataset (C. Smith, 2021) to investigate this question in the US Christian population, finding that material security in Wave 1 was indeed negatively associated with belief in God in Wave 4, even after accounting for potential confounders such as parents’ education and the ethnicity of respondents.

While testing the main hypotheses that material security during early adolescence would affect later belief in God, we remained agnostic about the timing of this influence as well as about the timing of the apostasy process. The hypotheses-testing approach using GORICA afforded this agnosticism because it allowed us to test the summed influence of material security in Wave 1 across the different data-collection waves, including the propagation of this influence via auto-regressive paths. Looking at the progression of the estimated effect sizes, however, we observed that the negative relationship between material security and belief in God became slightly stronger with each wave (see Table 3). This result might suggest that experiences during adolescence are indeed formative, although this conclusion is premature since our data did not allow us to test whether material security later in life (e.g., during young adulthood) might have a larger effect.

Overall, the results indicate that the predictive association between material security in childhood and belief in God in the early twenties is present but not particularly strong. Inspection of the raw data shows that the probability of a decrease in belief rises from approximately 5% among participants with low material security to about 11% among those with high material security. In the probit model, this corresponds to a latent mean difference of –0.63 between the least and most secure participants, which reflects a moderate effect size on the underlying latent response scale. It is further possible that the size of this effect is somewhat overestimated, however, as our sensitivity analyses using regression approaches rather than structural equation model yielded a slightly smaller overall coefficient (−0.04 vs −0.06). Using structural equation model allowed us to incorporate the longitudinal structure of the data, including autoregressive effects, and to model the hypothesized system of relationships (our DAG) in a single framework. Nevertheless, structural equation model requires stronger assumptions than a regression analysis, which might have biased our estimates. Yet, we believe that the similar results produced by these two approaches provide validity to our results. Similarly, further sensitivity checks revealed that when the ordinal nature of the outcome variable was ignored in maximum-likelihood models assuming multivariate normality, the association between material security and belief in God was further attenuated. While assuming normality in ordinal data violates several of the estimator’s core assumptions, the smaller effect in these models suggests that the observed relationship is sensitive to model choice.

Overall, while these results provide some support for the existential insecurity theory, the size of the association indicates that belief in God is likely influenced by additional factors, some of which we discuss in the next section.

### 4.1. Alternative pathways

We designed a DAG to lay out the potential pathways and eliminate alternative explanations, including an alternative mediation pathway between material security, education, and belief (Bruce, 2011; Schwadel, 2015, 2016; Vardy et al., 2022). In our controlled model, we also included relationships previously hypothesized in the extant literature, namely the positive effect of credible parental displays of religious faith on the fidelity of religious transmission (Henrich, 2009; Lanman & Buhrmester, 2017; Willard & Cingl, 2017).

Starting with the credibility-enhancing displays (CREDs), the theory predicts that children are sceptical cultural learners who evaluate the credibility of the model from which they learn (Henrich, 2009). In terms of religious beliefs, children should be more likely to imitate the beliefs of their parents if their parents credibly demonstrate them – for example, through costly religious behaviour. Thus, it may not be the direct effect of security on children that drives disbelief, but rather the absence of credible religious displays by their parents. We indeed found that parental ritual frequency positively predicted children’s belief in God, and the standardized effect size of this association surpassed the effect size we detected for material security (see Fig. 2 and Table S4 in the Supplementary Material). Although this result strongly supports the CREDs hypothesis, it should be interpreted with caution, as this relationship was not the focal one assessed in this paper and we did not control for other variables that could potentially confound it (Bulbulia, 2024; Rohrer et al., 2022). Yet, controlling for this association, we show that childhood material security itself shows predictive association with belief in God in young adulthood.

In a similar manner, we controlled for the mediating pathway between parents’ material security and its positive effect on children’s educational attainment, which might drive a decrease in belief in God. While the direct path between material security and belief in God (assumed by existential insecurity theory) survived this control, we found either no or only negligible association between education and belief in God. Compared to having no education or only a high school degree, graduating from a vocational school or a college/university did not significantly increase the probability of decreasing belief in God in Wave 4 (see Fig. 2 and Table S4 in the Supplementary Material for precise estimates). This result may be partly due to the fact that we included parents’ educational level and material security as predictors of children’s educational achievement, potentially suggesting that the previously detected relationship between degree attainment and belief in God may have been a spurious correlation. Nonetheless, we again note that this relationship was not the main focus of the current study, and that prior research has found associations between education and variables related to religiosity, though not necessarily belief in God itself (Schwadel, 2016).

Finally, we included participants’ income in Wave 3 to test whether material security later in life might explain belief in God in Wave 4, but we found no association. A speculative interpretation of this null finding is that material security may exert its formative influence specifically during early adolescence, although a longer timespan of data would be required to test this claim. Importantly, while some participants were already economically active in young adulthood, others still relied on parental support, further complicating the direct interpretation of this variable.

### 4.2. Limitations

These interpretations, as well as our main conclusion, are limited by the volume of missing data in our focal variables (see Fig. 1). To mitigate this limitation and increase statistical power (Woods et al., 2024), we imputed the missing data needed to test our hypotheses. However, while we assumed that a random process caused the observed attrition, it could be argued that the lack of belief in God motivated participants to continue in the study differently depending on their material security. In other words, insecure apostates might have been less likely to continue in the study than secure apostates. While we cannot exclude this possibility, the trends reported in Fig. 1A show that 70% of participants from low-material-security environments who reported no belief in God in Wave 2 continued to Wave 3 of data collection, and the same percentage continued to Wave 4. We believe that within the 30% who discontinued, not everyone dropped out because of a lack of interest in religion, which further mitigates the size of this potential bias. Moreover, our supplementary analysis reported in Section 5 of the Supplementary Material shows that while material security was positively associated with continued participation in Wave 4, this association did not differ between those who initially believed in God and those who did not.

Another major limitation of the current study was the operationalization of latent constructs in the NSYR dataset, which was typically restricted to a single variable, often measured only during one wave and on an ordinal scale. While we were primarily interested in the predictive association of material security during early adolescence (Wave 1) with later belief in God and, therefore, did not technically need to observe how material security experienced later in life relates to belief in God (assuming the effect of parental income attenuates and the apostasy process is usually complete), our tests would have been stronger if we had been able to model later associations between material security and belief in God during the maturation process, as well as potential cross-lagged relationships between belief in God and material security.

Further extensions of our approach might involve more direct assessments of the assumed causal relationship by using techniques that hypothetically intervene on the exposure variable, such as targeted minimum loss-based estimation (Bulbulia et al., 2024). While Granger causality statistically assesses the predictive relationship of material security for later belief in God, it does not imply causation. Employing counterfactual reasoning – asking what would happen if material security were manipulated – after controlling for confounding relationships, as in our paper, could provide an important test of the presumed causal relationship. Such a test would be unethical in the real world but is possible using hypothetical interventions on observational data.

Future studies may also wish to collect more nuanced data on psychological constructs such as well-being, perceived trust, and cooperative risks, in order to better assess the specific pressures (and their alleviation) that might drive belief in God. While we aimed to assess these relationships with the current dataset, using single variables to represent complex constructs (e.g., perceived health and trust in others) proved insufficient for our modelling endeavour (and thus the failure to fit the most complex preregistered model). Furthermore, there may be other important pressures we did not consider in the current paper (e.g., infant mortality, as suggested by Inglehart, 2020), although these pressures should ideally be contextualized and relevant for the population in question (i.e., we do not expect infant mortality to play a significant role in the religiosity of U.S. participants). Finally, our findings are limited by the cultural context we study: while the United States is a useful test case for secularization theories, we do not know whether our findings would generalize to other countries or religious traditions. Even within the United States, we limited our analyses to participants who initially professed belief; thus, our results cannot speak to how the dynamics between childhood material security and religiosity may unfold for individuals who converted to a religion later in life.

### 4.3. Conclusion

In conclusion, this study provided some support for a key assumption of existential security theory – albeit not without important limitations – namely, that material security during childhood is predictively associated with the later loss of belief in God. While based on a single sample of U.S. Christians, our results align with the broader observation that experiences during early adolescence predict people’s values, behaviours, and identities later in life. If adolescents perceive that vertically transmitted religious beliefs do not reflect their current environment (because of their existential security), they may choose not to invest in maintaining costly religious beliefs and practices, eventually contributing to the decline of religious systems, as observed in the secularization trends present in many societies. These results may therefore contribute to a broader understanding of how cultural systems adapt and transform in response to external pressures.

## Supporting information

Lang et al. supplementary material Lang et al. supplementary material

## Data Availability

Preregistration and materials are publicly available at the OSF: https://osf.io/s6nmg/. Analytical code and data can be found at GitHub: https://github.com/PetrPalisek/non-theism/.

## Supplementary material

The supplementary material for this article can be found at https://doi.org/10.1017/ehs.2025.10035.
