# Supplementary material for: The longitudinal associations of material security and belief in God in young Americans
Source: Evol Hum Sci. 2026 Jan 5;8:e2. doi: 10.1017/ehs.2025.10035 (PMC12895462; doi:10.1017/ehs.2025.10035)
Supplement: Lang et al. supplementary material [file S2513843X25100352sup001.docx]

**The longitudinal associations of material security and belief in God in young Americans**

**Supplementary Material**

Martin Lang,^1^* Petr Palíšek,^2^ and Radim Chvaja^3,4^

^1^ LEVYNA, Masaryk University, Brno, Czech Republic

^2^ Psychology Research Institute, Masaryk University, Brno, Czech Republic

^3^ Religion Programme, University of Otago, New Zealand

^4^ European Research University, Ostrava, Czech Republic

* Corresponding author: Martin Lang (martinlang@mail.muni.cz)

1. **Supplementary methods**
   1. Previous use of the NSYR dataset

The NSYR dataset covers several broad topics related to the role that religion plays in the lives of American youth, presenting an extremely rich resource for further analysis. As such, it has been previously utilized to investigate several research questions. For example, Smith (2021) examined how parental worship frequency and the importance of religion predict the difference in these variables in their children between Waves 1 and 4. Smith found that conservative parents were most successful in the religious socialization of their children, especially compared to parents with no political identification. Similarly, Perry and Longest (2019) investigated whether undergoing an initiation rite (e.g., bar/bat mitzvah, First Communion), as reported in Wave 1, predicts disaffiliation from a religious tradition in Wave 4, finding that partaking in these rites did not predict religiosity at the last wave but predicted a lower probability of disaffiliation from a religious tradition. Hardie and colleagues (2016) compared the change in religious attendance between Wave 1 and Wave 2, observing a 10% decline in attendance. While initiating a sexual relationship between the two waves predicted a decline in attendance, living in an environment supporting ritual participation or experiencing a traumatic life event acted as a buffer against this decline. Mikoski and Olson (2021) used the first-wave data to show that children of parents living in counties where their religious tradition is in the majority report higher values on a religiosity scale compared to children whose parents affiliate with traditions that are in the minority. Analyzing the effects of higher education on religiosity, Schwadel (2016) found that obtaining a bachelor’s degree was associated with a decrease in the frequency of prayer, religious certainty, and belief but had no effect on the frequency of church attendance or personal religiosity. Finally, Schwadel (2008) used the first wave data to divide participants into poor and non-poor families based on parents’ income and showed that poor teenagers are less likely to partake in organized religious activities (church attendance, Sunday school) but are more likely to engage in personal religious activities (prayer, scripture reading). While this last research is close to our aim in the current paper, it uses only Wave 1 data and lacks the longitudinal dimension that may help us assess the Granger causality between material security in childhood and religious belief in young adulthood.

1. **Supplementary hypotheses tests**

When thinking about our analyses during pre-registration, we aimed to also test specific mediating mechanisms that might track the exogenous pressures fostering beliefs—namely on cooperation (operationalized as a lack of trustworthy relationships) and health (operationalized as health problems). These analyses were meant to help us distinguish between these two major functions of religion as described in the main text. We also planned to investigate whether the purported decline in belief in God may be preceded by a decline in religious practice, namely the frequency of service attendance and prayer. See Figure S1.


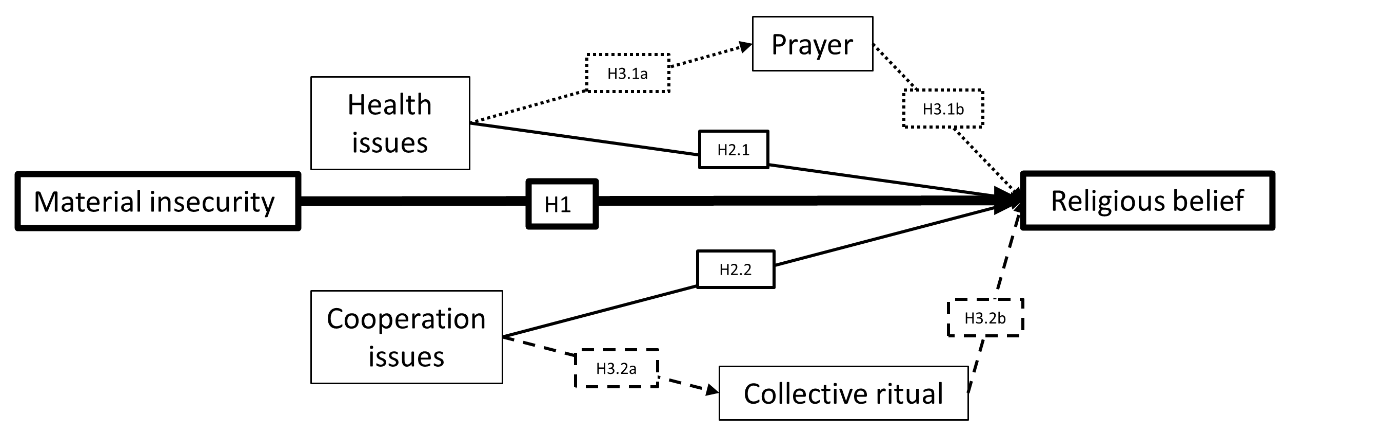


**Figure S1| Overview of the mediating mechanisms we originally planned to test using the NSYR data set.** All displayed pathways are assumed to be positive; that is, material insecurity increases belief, health issues increase prayer frequency, etc.

### Based on the review of the theory, we pre-registered the following hypotheses:

Existential insecurity theory

H1: Higher parental material security in Wave 1 predicts participants’ lower self-reported belief in God in Wave 4.

Specific exogenous pressures affecting belief in God:

H2.1: Better self-reported health in Wave 2 predicts participants’ lower self-reported belief in God in Wave 4.

H2.2: Higher trust toward others in Wave 2 predicts participants’ lower self-reported belief in God in Wave 4.

Mechanisms of religious systems mediating the effects of exogenous pressures:

H3.1: The negative effect of self-reported health in Wave 2 on self-reported belief in God in Wave 4 is mediated by the frequency of prayer in Wave 3.

H3.2: The negative effect of trust in others in Wave 2 on self-reported belief in God in Wave 4 is mediated by the frequency of religious service attendance in Wave 3.

To test these hypotheses, we operationalized the pressures as follows: First, the existential insecurity paths between material security and subjects’ self-assessed health and the cooperative affordances of the subjects’ networks are captured by variables health and trust in Wave 2. Health is a single item inquiring about the participant’s self-reported health on a 1-5 scale from “excellent” to “poor.” Trust is a single item asking about the generalized trust assessment of the individual towards others. The individual chooses between stances “most people can be trusted,” “can’t be too careful,” or “depends.” Second, since the effect of health (Wave 2) and trust (Wave 2) on disbelief in God (Wave 4) might be mediated by the frequency of personal prayer and the degree of participation in collective rituals, we included variables prayer and attendance in Wave 3. Prayer asks how often participants pray alone (ranging from “never” to “multiple times a day”). Attendance asks how often participants attend religious services other than weddings, funerals, and baptisms (ranging from “never” to “multiple times a week”).

Using these variables, we fitted a ‘full’ model that included both the direct path of interest between material security in Wave 1 and belief in God in Waves 2-4, and also the pathways from exogenous pressures via ritual practice. That is, compared to the controls model reported in the main text, the full model includes explanatory paths hypothesized by H2 and H3. However, this model did not converge, suggesting our specification of the model was flawed or the data at hand were not sufficient to fit such a complex model. To help the model converge, we deleted the paths from material security in Wave 1 to collective and personal ritual frequency in Waves 2-3, as well as delete covariation of personal and collective ritual in Wave 4 with belief in God in Wave 4. None of the changes directly affect our hypothesized paths of importance; yet these changes allow the model to be agnostic about the relationship between material security and the frequency of collective and personal rituals since these effects often covary and likely caused modelling issues when included in the same model. On the other hand, these changes still allow us to explore whether material security in Wave 1 affects trust in Wave 2 and whether the effect of trust on belief in God in Wave 4 is mediated by collective ritual frequency in Wave 3—H3.2 (likewise for health and personal ritual frequency—H3.1).

For the sake of consistency, we followed the changes made to the specification of the model reported in the manuscript by including correlations between dummy variables created from the variable categorizing religious affiliation and adding paths from parental ritual to belief in God in each wave. In contrast to the reported model, we did not freely estimate the correlation between Income in Wave 3 and the dummy variable for college education because doing so severely inflated the standard errors for affected regression coefficients. After inspecting the residual matrix, we also freely estimated the correlations between parental ritual and collective and personal ritual to eliminate stark local misfit. Nevertheless, even after this model-specification change and the additional corrections based on the residual matrix, the fit indices exhibited inadequate fit to the data: χ^2^(251) = 1801.46, D = 1.29, RMSEA = .047, _90%_CI = [.045; .049], SRMR = .064, TLI = 0.510. Though it can be argued that RMSEA of the null model is low (.066), thus making TLI uninformative, SRMR indicates that the model reproduces the observed variance-covariance matrix inaccurately. *Given this inadequacy, in combination with poor convergence properties, we report the planned analysis below, but caution readers that the model may not be adequate for the data at hand and the assumed causal structure may be misspecified.*

As in our other models, material security in Wave 1 predicted lower belief in God in Wave 4 (cumulative b = -0.10, _95%_CI [-0.17, -0.04], p = .002). Regarding H2, we observed an opposite effect than predicted for self-reported health (b = 0.41 with 95%CI [0.24; 0.57], p < .001). For trust, we observed a negative effect (b = -0.33 with _95%_CI [-0.50; -0.16], p < .001). Finally, testing H3 revealed that self-reported health in Wave 2 had a weak and negative indirect effect on belief in God in Wave 4 via personal ritual frequency in Wave 3: b* = -0.05 with _95%_CI [-0.08; -0.02], p = .001. However, note that while in the predicted direction, this effect is driven by the model-implied positive effect of health on personal ritual frequency (b* = 0.20), which in turn predicts lower belief in God (b* = -.26). It is important to reiterate that these counterintuitive estimates are likely not accurate due to the model’s overall performance. Self-reported trust in Wave 2, on the other hand, had a weak and positive effect on belief in God in Wave 4 via collective ritual frequency in Wave 3: b* = 0.06 with _95%_CI [0.03; 0.08], p < .001. See Table S1 for model estimates and Table S5 for full model parameters.

| **Table S1 \|** Full model estimates (pooled) | | | | | |
| --- | --- | --- | --- | --- | --- |
|  |  | b* | SE | z | p |
| *Auto-regressive paths* | |  |  |  |  |
| Belief in God1 -> Belief in God 2 | | 0.49 | 0.06 | 8.68 | <.001 |
| Belief in God2 -> Belief in God 3 | | 0.66 | 0.11 | 5.88 | <.001 |
| Belief in God3 -> Belief in God 4 | | 0.90 | 0.15 | 5.97 | <.001 |
| *Lagged paths* | |  |  |  |  |
| Material Security1 -> Belief in God2 | | 0.004 | 0.03 | 0.13 | .896 |
| Material Security1 -> Belief in God3 | | -0.02 | 0.03 | -0.74 | .462 |
| Material Security1 -> Belief in God4 | | -0.14 | 0.03 | -4.27 | < .001 |
| *Hypothesis2* | |  |  |  |  |
| Health2 -> Belief in God4 | | 0.24 | 0.05 | 5.17 | < .001 |
| Trust2 -> Belief in God4 | | -0.20 | 0.06 | -3.50 | < .001 |
| *Hypothesis3* | |  |  |  |  |
| Health2 -> Private Ritual3 | | 0.20 | 0.02 | 8.21 | < .001 |
| Private Ritual3 -> Belief in God4 | | -0.26 | 0.07 | -3.67 | < .001 |
| Health2 -> Private Ritual3 -> Belief in God4 | | -0.05 | 0.02 | -3.31 | .001 |
| Trust2 -> Collective Ritual3 | | -0.18 | 0.03 | -6.70 | <.001 |
| Collective Ritual3 -> Belief in God4 | | -0.30 | 0.07 | -3.67 | <.001 |
| Trust 2 -> Collective Ritual3 -> Belief in God4 | | 0.06 | 0.01 | 4.10 | < .001 |
| *Note.* b* = standardized regression coefficient; SE = standard error of b; z = z-statistic value; p = p-value for the z-statistic. Numbers next to variables denote data-collection waves. In the full model, we used the same set of controls as described in Table 3. | | | | | |

After fitting the full model, we used GORICA to estimate the relative support for the planned hypotheses, finding that the effect hypothesized in H1 is 27 times more likely than the complement of this model. H2.1 had overwhelming support for the complement, indicating that the observed effect is in the opposite direction than expected, while support for H2.2 was overwhelming. The support for partial mediation was weak to none for H3.1. The complement was preferred for H3.2. See Table S2 below for details.

| **Table S2 \|** Informative hypotheses’ tests | | | | | | |
| --- | --- | --- | --- | --- | --- | --- |
| Model | Hypothesis | LL hypo | LL comp | PW | GW ratio | Support |
| *Baseline* |  |  |  |  |  |  |
|  | H1 | 20.53 | 11.33 | 0.5 | 9854 | overwhelming |
| *Controls* |  |  |  |  |  |  |
|  | H1 | 21.90 | 19.08 | 0.5 | 16.90 | weak |
| *Full* |  |  |  |  |  |  |
|  | H1 | 41.48 | 38.18 | 0.5 | 27 | strong |
|  | H2.1 | 28.13 | 41.48 | 0.5 | 0 | none |
|  | H2.2 | 41.48 | 35.36 | 0.5 | 453 | overwhelming |
|  | H3.1 | 41.48 | 41.20 | 0.61 | 2.09 | none |
|  | H3.2 | 32.97 | 41.48 | 0.67 | 0 | none |
| *Note:* LL hypo = log-likelihood of H1; LL comp = log-likelihood of the complement; PW = hypothesis penalty weight (i.e., the ratio of the parameter space covered by the hypothesis); GW ratio = GORICA weight ratio (i.e., the relative support of the hypothesis compared to the complement); support = verbal interpretation of the results. | | | | | | |

1. **Complete model estimates**

| **Table S3 \| Estimates from the baseline model** | | | | | | | | |
| --- | --- | --- | --- | --- | --- | --- | --- | --- |
| *Path name* | | | *b* | *SE* | *z* | *p* | *Low CI* | *Up CI* |
| Bel God2 | ~ | Bel God1 | 0.00 |  |  |  |  |  |
| Bel God2 | ~ | Mat Sec1 | 0.34 | 0.06 | 6.06 | < 0.001 | 0.23 | 0.45 |
| Bel God3 | ~ | Bel God2 | -0.03 | 0.02 | -1.96 | 0.05 | -0.07 | 0.00 |
| Bel God3 | ~ | Mat Sec1 | 0.32 | 0.04 | 8.46 | < 0.001 | 0.24 | 0.39 |
| Bel God4 | ~ | Bel God3 | -0.04 | 0.02 | -2.77 | 0.01 | -0.08 | -0.01 |
| Bel God4 | ~ | Mat Sec1 | 0.33 | 0.04 | 9.11 | < 0.001 | 0.26 | 0.40 |
| *Note.* We only report regression path estimates. For other model parameters see the Supplementary R code. Numbers next to each variable denote data-collection wave. Full variable names: Bel God = Belief in God; Mat Sec = material security. Full parameter names: b = Unstandardized regression coefficient estimate; SE = Standard error; z = z-statistic; p = p-value; Low CI = lower 95% Confidence Interval; Up CI = Upper 95% Confidence Interval. | | | | | | | | |

| **Table S4 \| Estimates from the model with controls.** | | | | | | | | |
| --- | --- | --- | --- | --- | --- | --- | --- | --- |
| *Path name* | | | *b* | *SE* | *z* | *p* | *Low CI* | *Up CI* |
| BiG1 | ~ | Male | 0.24 | 0.08 | 2.82 | < . 001 | 0.07 | 0.40 |
| BiG1 | ~ | BlackProt | 0.52 | 0.23 | 2.29 | 0.02 | 0.07 | 0.96 |
| BiG1 | ~ | Catholic | -0.27 | 0.10 | -2.84 | < . 001 | -0.46 | -0.09 |
| BiG1 | ~ | MainProt | -0.14 | 0.13 | -1.09 | 0.27 | -0.39 | 0.11 |
| BiG1 | ~ | BlackE | 0.44 | 0.22 | 1.99 | 0.05 | 0.01 | 0.87 |
| BiG1 | ~ | LatinxE | 0.25 | 0.14 | 1.72 | 0.09 | -0.04 | 0.53 |
| BiG1 | ~ | OtherE | -0.14 | 0.17 | -0.85 | 0.39 | -0.47 | 0.19 |
| BiG1 | ~ | ParRit | 0.34 | 0.04 | 8.22 | < . 001 | 0.26 | 0.42 |
| BiG2 | ~ | Male | 0.29 | 0.10 | 3.07 | < . 001 | 0.11 | 0.48 |
| BiG2 | ~ | BlackProt | 0.50 | 0.29 | 1.72 | 0.08 | -0.07 | 1.07 |
| BiG2 | ~ | Catholic | -0.38 | 0.12 | -3.14 | < . 001 | -0.61 | -0.14 |
| BiG2 | ~ | MainProt | -0.34 | 0.15 | -2.23 | 0.03 | -0.64 | -0.04 |
| BiG2 | ~ | BlackE | 0.52 | 0.27 | 1.96 | 0.05 | 0.00 | 1.04 |
| BiG2 | ~ | LatinxE | 0.44 | 0.19 | 2.27 | 0.02 | 0.06 | 0.82 |
| BiG2 | ~ | OtherE | 0.26 | 0.23 | 1.11 | 0.27 | -0.19 | 0.71 |
| BiG2 | ~ | ParRit | 0.43 | 0.05 | 8.63 | < . 001 | 0.33 | 0.52 |
| BiG3 | ~ | Male | 0.38 | 0.10 | 3.67 | < . 001 | 0.18 | 0.58 |
| BiG3 | ~ | BlackProt | 0.22 | 0.33 | 0.66 | 0.51 | -0.42 | 0.85 |
| BiG3 | ~ | Catholic | -0.23 | 0.11 | -2.04 | 0.04 | -0.45 | -0.01 |
| BiG3 | ~ | MainProt | -0.42 | 0.14 | -2.96 | < . 001 | -0.69 | -0.14 |
| BiG3 | ~ | BlackE | 1.10 | 0.30 | 3.69 | < . 001 | 0.52 | 1.68 |
| BiG3 | ~ | LatinxE | 0.27 | 0.19 | 1.44 | 0.15 | -0.10 | 0.64 |
| BiG3 | ~ | OtherE | 0.21 | 0.21 | 0.99 | 0.32 | -0.20 | 0.61 |
| BiG3 | ~ | ParRit | 0.37 | 0.05 | 7.52 | < . 001 | 0.28 | 0.47 |
| BiG4 | ~ | Age | 0.09 | 0.05 | 1.69 | 0.09 | -0.01 | 0.20 |
| BiG4 | ~ | Male | 0.40 | 0.10 | 4.07 | < . 001 | 0.20 | 0.59 |
| BiG4 | ~ | BlackProt | 0.21 | 0.34 | 0.62 | 0.54 | -0.45 | 0.87 |
| BiG4 | ~ | Catholic | -0.16 | 0.12 | -1.41 | 0.16 | -0.39 | 0.06 |
| BiG4 | ~ | MainProt | -0.27 | 0.14 | -1.88 | 0.06 | -0.55 | 0.01 |
| BiG4 | ~ | BlackE | 0.78 | 0.31 | 2.53 | 0.01 | 0.18 | 1.38 |
| BiG4 | ~ | LatinxE | 0.54 | 0.19 | 2.79 | 0.01 | 0.16 | 0.91 |
| BiG4 | ~ | OtherE | -0.04 | 0.20 | -0.21 | 0.84 | -0.44 | 0.36 |
| BiG4 | ~ | Inc3 | 0.04 | 0.07 | 0.56 | 0.57 | -0.09 | 0.17 |
| BiG4 | ~ | ParRit | 0.22 | 0.05 | 4.36 | < . 001 | 0.12 | 0.32 |
| BiG4 | ~ | College | -0.09 | 0.14 | -0.67 | 0.50 | -0.37 | 0.18 |
| BiG4 | ~ | AAVOC | -0.18 | 0.19 | -0.97 | 0.33 | -0.54 | 0.18 |
| BiG4 | ~ | PST | -0.07 | 0.06 | -1.15 | 0.25 | -0.19 | 0.05 |
| BiG2 | ~ | BiG1 | 0.31 | 0.07 | 4.26 | < . 001 | 0.17 | 0.45 |
| BiG2 | ~ | MS1 | -0.01 | 0.02 | -0.60 | 0.55 | -0.05 | 0.02 |
| BiG3 | ~ | BiG2 | 0.26 | 0.05 | 5.60 | < . 001 | 0.17 | 0.35 |
| BiG3 | ~ | MS1 | -0.03 | 0.02 | -1.67 | 0.09 | -0.06 | 0.00 |
| BiG4 | ~ | BiG3 | 0.27 | 0.04 | 6.07 | < . 001 | 0.18 | 0.36 |
| BiG4 | ~ | MS1 | -0.06 | 0.02 | -3.40 | < . 001 | -0.10 | -0.03 |
| MS1 | ~ | ParCollege | 3.28 | 0.13 | 24.72 | < . 001 | 3.02 | 3.54 |
| MS1 | ~ | ParAAVOC | 1.25 | 0.15 | 8.50 | < . 001 | 0.96 | 1.54 |
| MS1 | ~ | BlackE | -1.57 | 0.14 | -11.55 | < . 001 | -1.83 | -1.30 |
| MS1 | ~ | LatinxE | -1.25 | 0.17 | -7.39 | < . 001 | -1.58 | -0.92 |
| MS1 | ~ | OtherE | -0.21 | 0.21 | -1.01 | 0.31 | -0.63 | 0.20 |
| BlackProt | ~ | BlackE | 0.70 | 0.02 | 32.34 | < . 001 | 0.66 | 0.75 |
| Catholic | ~ | LatinxE | 0.42 | 0.05 | 7.96 | < . 001 | 0.32 | 0.52 |
| BlackProt | ~ | OtherE | 0.03 | 0.03 | 1.17 | 0.24 | -0.02 | 0.08 |
| MainProt | ~ | OtherE | -0.05 | 0.03 | -1.80 | 0.07 | -0.11 | 0.00 |
| Catholic | ~ | OtherE | 0.22 | 0.04 | 5.48 | < . 001 | 0.14 | 0.29 |
| College | ~ | ParCollege | 0.14 | 0.04 | 3.64 | < . 001 | 0.06 | 0.21 |
| College | ~ | ParAAVOC | -0.01 | 0.03 | -0.19 | 0.85 | -0.06 | 0.05 |
| College | ~ | Age | 0.03 | 0.01 | 3.09 | < . 001 | 0.01 | 0.05 |
| College | ~ | MS1 | 0.05 | 0.01 | 6.12 | < . 001 | 0.03 | 0.07 |
| AAVOC | ~ | ParCollege | 0.02 | 0.02 | 1.07 | 0.29 | -0.02 | 0.07 |
| AAVOC | ~ | ParAAVOC | 0.03 | 0.02 | 1.32 | 0.19 | -0.01 | 0.07 |
| AAVOC | ~ | Age | 0.01 | 0.01 | 0.82 | 0.41 | -0.01 | 0.02 |
| AAVOC | ~ | MS1 | 0.00 | 0.00 | -1.51 | 0.13 | -0.01 | 0.00 |
| *Note.* We only report regression path estimates. For other model parameters see the Supplementary R code. Numbers next to each variable denote data-collection wave. Reference categories for categorical predictors: Gender – Women; Ethnicity: White; Religious tradition: Conservative Protestantism; Education: no/high school. Full variable names: Bel God = Belief in God; Prot = Protestant; Eth = Ethnicity; Par = Parental; Rit = Ritual; AAVOC = Associate degree/Vocational school; Mat Sec = material security. Full parameter names: b = unstandardized regression coefficient estimate; SE = Standard error; z = z-statistic; p = p-value; Low CI = lower 95% Confidence Interval; Up CI = Upper 95% Confidence Interval. | | | | | | | | |

| **Table S5 \| Estimates from the full model.** | | | | | | | | |
| --- | --- | --- | --- | --- | --- | --- | --- | --- |
| *Path name* | | | *b* | *SE* | *z* | *p* | *Low CI* | *Up CI* |
| Bel God1 | ~ | Men1 | 0.22 | 0.08 | 2.82 | < .001 | 0.07 | 0.37 |
| Bel God1 | ~ | Black Prot1 | 0.54 | 0.21 | 2.53 | 0.01 | 0.12 | 0.96 |
| Bel God1 | ~ | Catholic1 | -0.24 | 0.09 | -2.62 | 0.01 | -0.43 | -0.06 |
| Bel God1 | ~ | Main Prot1 | -0.12 | 0.12 | -0.98 | 0.33 | -0.35 | 0.12 |
| Bel God1 | ~ | Black Eth1 | 0.37 | 0.21 | 1.79 | 0.07 | -0.03 | 0.77 |
| Bel God1 | ~ | Latinx Eth1 | 0.23 | 0.13 | 1.73 | 0.08 | -0.03 | 0.50 |
| Bel God1 | ~ | Other Eth1 | -0.14 | 0.16 | -0.87 | 0.38 | -0.44 | 0.17 |
| Bel God1 | ~ | Par Rit1 | 0.27 | 0.04 | 6.76 | < .001 | 0.19 | 0.35 |
| Bel God2 | ~ | Men1 | 0.19 | 0.09 | 2.12 | 0.03 | 0.01 | 0.37 |
| Bel God2 | ~ | Black Prot1 | 0.16 | 0.28 | 0.58 | 0.56 | -0.39 | 0.72 |
| Bel God2 | ~ | Catholic1 | -0.27 | 0.11 | -2.42 | 0.02 | -0.49 | -0.05 |
| Bel God2 | ~ | Main Prot1 | -0.29 | 0.15 | -2.00 | 0.05 | -0.58 | -0.01 |
| Bel God2 | ~ | Black Eth1 | 0.45 | 0.26 | 1.73 | 0.08 | -0.06 | 0.97 |
| Bel God2 | ~ | Latinx Eth1 | 0.35 | 0.19 | 1.85 | 0.06 | -0.02 | 0.73 |
| Bel God2 | ~ | Other Eth1 | 0.29 | 0.22 | 1.35 | 0.18 | -0.13 | 0.72 |
| Bel God2 | ~ | Par Rit1 | 0.29 | 0.06 | 4.76 | < .001 | 0.17 | 0.41 |
| Bel God3 | ~ | Men1 | 0.21 | 0.12 | 1.71 | 0.09 | -0.03 | 0.44 |
| Bel God3 | ~ | Black Prot1 | -0.07 | 0.35 | -0.21 | 0.83 | -0.75 | 0.61 |
| Bel God3 | ~ | Catholic1 | 0.06 | 0.12 | 0.46 | 0.65 | -0.18 | 0.29 |
| Bel God3 | ~ | Main Prot1 | -0.13 | 0.15 | -0.89 | 0.37 | -0.43 | 0.16 |
| Bel God3 | ~ | Black Eth1 | 0.76 | 0.31 | 2.42 | 0.02 | 0.14 | 1.37 |
| Bel God3 | ~ | Latinx Eth1 | -0.01 | 0.20 | -0.05 | 0.96 | -0.40 | 0.38 |
| Bel God3 | ~ | Other Eth1 | 0.09 | 0.21 | 0.45 | 0.65 | -0.31 | 0.50 |
| Bel God3 | ~ | Par Rit1 | 0.52 | 0.08 | 6.49 | < .001 | 0.36 | 0.67 |
| Bel God4 | ~ | Age4 | 0.08 | 0.05 | 1.65 | 0.10 | -0.02 | 0.18 |
| Bel God4 | ~ | Men1 | 0.09 | 0.12 | 0.72 | 0.47 | -0.15 | 0.33 |
| Bel God4 | ~ | Black Prot1 | 0.11 | 0.38 | 0.30 | 0.77 | -0.63 | 0.86 |
| Bel God4 | ~ | Catholic1 | -0.07 | 0.13 | -0.53 | 0.60 | -0.32 | 0.18 |
| Bel God4 | ~ | Main Prot1 | -0.07 | 0.17 | -0.44 | 0.66 | -0.41 | 0.26 |
| Bel God4 | ~ | Black Eth1 | -0.19 | 0.39 | -0.48 | 0.63 | -0.96 | 0.58 |
| Bel God4 | ~ | Latinx Eth1 | 0.26 | 0.21 | 1.25 | 0.21 | -0.15 | 0.68 |
| Bel God4 | ~ | Other Eth1 | -0.18 | 0.21 | -0.88 | 0.38 | -0.59 | 0.22 |
| Bel God4 | ~ | Income3 | 0.05 | 0.06 | 0.83 | 0.41 | -0.07 | 0.18 |
| Bel God4 | ~ | Par Rit1 | 0.25 | 0.08 | 3.24 | < .001 | 0.10 | 0.41 |
| Bel God4 | ~ | College4 | 0.03 | 0.12 | 0.26 | 0.80 | -0.21 | 0.27 |
| Bel God4 | ~ | AAVOC4 | -0.13 | 0.16 | -0.85 | 0.40 | -0.44 | 0.18 |
| Bel God4 | ~ | Par Stress1 | -0.07 | 0.06 | -1.09 | 0.28 | -0.19 | 0.05 |
| Bel God2 | ~ | Bel God1 | 0.67 | 0.10 | 6.57 | < .001 | 0.47 | 0.87 |
| Bel God2 | ~ | Mat Sec1 | 0.00 | 0.02 | 0.15 | 0.88 | -0.03 | 0.03 |
| Bel God3 | ~ | Bel God2 | 0.73 | 0.13 | 5.41 | < .001 | 0.47 | 0.99 |
| Bel God3 | ~ | Mat Sec1 | -0.01 | 0.02 | -0.77 | 0.44 | -0.05 | 0.02 |
| Bel God3 | ~ | Health2 | 0.35 | 0.06 | 5.55 | < .001 | 0.23 | 0.47 |
| Bel God3 | ~ | Trust2 | -0.32 | 0.08 | -4.03 | < .001 | -0.47 | -0.16 |
| Bel God3 | ~ | Priv Rit2 | -0.18 | 0.08 | -2.08 | 0.04 | -0.34 | -0.01 |
| Bel God3 | ~ | Coll Rit2 | -0.13 | 0.05 | -2.36 | 0.02 | -0.24 | -0.02 |
| Bel God4 | ~ | Bel God3 | 0.86 | 0.19 | 4.63 | < .001 | 0.50 | 1.23 |
| Bel God4 | ~ | Mat Sec1 | -0.08 | 0.02 | -3.96 | < .001 | -0.12 | -0.04 |
| Bel God4 | ~ | Health2 | 0.05 | 0.08 | 0.64 | 0.52 | -0.11 | 0.21 |
| Bel God4 | ~ | Trust2 | -0.06 | 0.08 | -0.74 | 0.46 | -0.20 | 0.09 |
| Bel God4 | ~ | Health3 | 0.10 | 0.07 | 1.56 | 0.12 | -0.03 | 0.23 |
| Bel God4 | ~ | Priv Rit3 | -0.28 | 0.08 | -3.35 | < .001 | -0.44 | -0.11 |
| Bel God4 | ~ | Coll Rit3 | -0.31 | 0.06 | -4.87 | < .001 | -0.43 | -0.18 |
| Mat Sec1 | ~ | Par College1 | 3.30 | 0.14 | 24.24 | < .001 | 3.03 | 3.57 |
| Mat Sec1 | ~ | Par AAVOC1 | 1.12 | 0.15 | 7.60 | < .001 | 0.83 | 1.42 |
| Mat Sec1 | ~ | Black Eth1 | -1.58 | 0.14 | -11.62 | < .001 | -1.84 | -1.31 |
| Mat Sec1 | ~ | Latinx Eth1 | -1.20 | 0.17 | -6.94 | < .001 | -1.53 | -0.86 |
| Mat Sec1 | ~ | Other Eth1 | -0.22 | 0.21 | -1.02 | 0.31 | -0.64 | 0.20 |
| BlackProt1 | ~ | Black Eth1 | 0.71 | 0.02 | 32.32 | < .001 | 0.66 | 0.75 |
| Catholic1 | ~ | Latinx Eth1 | 0.43 | 0.05 | 7.87 | < .001 | 0.32 | 0.53 |
| BlackProt1 | ~ | Other Eth1 | 0.03 | 0.03 | 1.18 | 0.24 | -0.02 | 0.08 |
| MainProt1 | ~ | Other Eth1 | -0.05 | 0.03 | -1.80 | 0.07 | -0.11 | 0.00 |
| Catholic1 | ~ | Other Eth1 | 0.22 | 0.04 | 5.46 | < .001 | 0.14 | 0.29 |
| College4 | ~ | Par College1 | 0.08 | 0.04 | 2.30 | 0.02 | 0.01 | 0.16 |
| College4 | ~ | Par AAVOC1 | -0.02 | 0.03 | -0.65 | 0.51 | -0.08 | 0.04 |
| College4 | ~ | Age4 | 0.03 | 0.01 | 3.05 | < .001 | 0.01 | 0.05 |
| College4 | ~ | Mat Sec1 | 0.06 | 0.01 | 6.19 | < .001 | 0.04 | 0.08 |
| AAVOC4 | ~ | Par College1 | 0.02 | 0.02 | 1.01 | 0.31 | -0.02 | 0.07 |
| AAVOC4 | ~ | Par AAVOC1 | 0.03 | 0.02 | 1.26 | 0.21 | -0.01 | 0.07 |
| AAVOC4 | ~ | Age | 0.01 | 0.01 | 0.82 | 0.41 | -0.01 | 0.02 |
| AAVOC4 | ~ | Mat Sec1 | 0.00 | 0.00 | -1.35 | 0.18 | -0.01 | 0.00 |
| Income3 | ~ | Mat Sec1 | 0.00 | 0.01 | 0.33 | 0.74 | -0.01 | 0.02 |
| Par Rit1 | ~ | Par College1 | 0.26 | 0.06 | 4.62 | < .001 | 0.15 | 0.37 |
| Par Rit1 | ~ | Par AAVOC1 | 0.02 | 0.06 | 0.27 | 0.79 | -0.10 | 0.13 |
| Priv Rit3 | ~ | Priv Rit2 | 0.80 | 0.02 | 32.44 | < .001 | 0.75 | 0.84 |
| Priv Rit3 | ~ | Health2 | 0.32 | 0.04 | 7.31 | < .001 | 0.24 | 0.41 |
| Priv Rit3 | ~ | Trust2 | -0.43 | 0.06 | -7.28 | < .001 | -0.55 | -0.31 |
| Coll Rit3 | ~ | Coll Rit2 | 0.58 | 0.03 | 19.16 | < .001 | 0.52 | 0.63 |
| Coll Rit3 | ~ | Health2 | 0.44 | 0.05 | 8.66 | < .001 | 0.34 | 0.53 |
| Coll Rit3 | ~ | Trust2 | -0.30 | 0.05 | -5.44 | < .001 | -0.41 | -0.19 |
| Health2 | ~ | Mat Sec1 | 0.06 | 0.01 | 6.76 | < .001 | 0.04 | 0.08 |
| Health3 | ~ | Health2 | 0.54 | 0.03 | 20.98 | < .001 | 0.49 | 0.59 |
| Trust2 | ~ | Mat Sec1 | 0.11 | 0.01 | 9.60 | < .001 | 0.09 | 0.13 |

*Note.* We only report regression path estimates. For other model parameters see the Supplementary R code. Numbers next to each variable denote data-collection wave. Reference categories for categorical predictors: Gender – Women; Ethnicity: White; Religious tradition: Conservative Protestantism; Education: no/high school. Full variable names: Bel God = Belief in God; Prot = Protestant; Eth = Ethnicity; Par = Parental; Rit = Ritual; AAVOC = Associate degree/Vocational school; Mat Sec = material security. Full parameter names: b = unstandardized regression coefficient estimate; SE = Standard error; z = z-statistic; p = p-value; Low CI = lower 95% Confidence Interval; Up CI = Upper 95% Confidence Interval.

1. **Supplementary sensitivity checks**

As an initial check (non-preregistered, requested by a reviewer), we fit a regression model predicting belief in God in Wave 4 by material security in Wave 1. The goal of this analysis was to provide a rough benchmark for the association of material security on belief in God in Wave 4. Too do so, we used the mice-imputed datasets and pooled the model estimates according to Rubin’s Rules (Rubin, 1987). Note that due to the outcome’s ordinal nature and asymmetric category frequencies, we used the ordinal regression with the complementary log-log (clog-log) link function rather than other viable alternatives such as log-log, probit, or logit. The clog-log link function minimized the mean AIC of our models more compared to using the probit and log-log link functions; and while the log link had lower mean AIC, the difference was small, and complementary log-log was preferred due to the asymmetricity of categories of our outcome variable. Details are available in the Supplementary R code.

This procedure showed a negative association between material security and belief in God in Wave 4 when disregarding any additional variables (b = -0.08, _95%_CI [-0.11; -0.05], p < .001). While this initial model supported the predicted relationship between material security and belief in God, this association is rather weak. Following reviewer’s suggestion, we also show the results for ordinal regression with appropriate controls (the setup mimics the second SEM model described further below). In this model, the negative association remained albeit with weaker effect size and non-significant p-value: b = -0.04, _95%_CI [-0.08 ; 0.003], p = .071. Note these analyses are not to be interpreted as our main results, rather, they show that the predicted association can be detected with simpler regression methods compared to SEM, and it is of a similar magnitude to the SEM estimate. Nevertheless, as pre-registered, our main strategy relied on an SEM that estimated the cumulative association between material security and belief in God across all time waves.

We provide further pre-registered sensitivity tests reported in Table S6. While most of these models support the conclusions drawn in the main text, models with controls using maximum-likelihood estimator do not show such a support. However, our main analytical strategy relied on ordinal models estimated with weighted least square mean and variance adjusted estimator (WLSMV) that correctly reflect the fact that our main outcome variable had only three ordered categories, which severely undermines the assumptions of multivariate normal distribution required for maximum-likelihood models (Rhemtulla et al., 2012). Thus, inappropriately assuming normality could lead to Type II errors, which we avoid by using the WLSMV estimator.

Furthermore, as pre-registered, we also fit 11 nested models each representing one hypothesis through corresponding parameter restrictions (i.e., fixing paths to zero or allowing them to be freely estimated), and compared their relative fit. The best fitting models, according to RMSEA, TLI and SRMR, was the full model with paths from hypotheses other than H1 fixed to zero (see Table S7). Nevertheless, all nested models fit equally well as the differences in fit index values are negligible. This procedure would have been more conclusive with information criteria such as AIC and BIC which are not available in ordinal SEM *lavaan* models.

| **Table S6 \| Sensitivity analysis of H1 across various modeling approaches.** | | | | |  |  |  |  |
| --- | --- | --- | --- | --- | --- | --- | --- | --- |
| Specification | Method | LL hypothesis | LL complement | gw ratio | | 5% ; 50% ; 95% null | 5% ; 50% ; 95% obs | support |
| *Baseline* |  |  |  |  | |  |  |  |
|  | WLSMV (mice) | 20.53 | 11.33 | 9854 | | [0.22 ; 1 ; 4.06] | [39.69; 1.1e04; 6.8e07] | overwhelming |
|  | WLSMV (pairwise) | 19.69 | 11.57 | 3382 | | [.31 ; 1 ; 3.91] | [18.04 ; 2989; 7.8e06] | overwhelming |
|  | ML (mice) | 21.79 | 16.78 | 150 | | [0.26 ; 1.00 ; 3.85] | [3.45 ; 180; 1.4e5] | overwhelming |
|  | FIML | 20.71 | 13.41 | 1487 | | [0.26 ; 1.00 ; 3.64] | [13.27 ; 1996; 2.4e6] | overwhelming |
|  |  |  |  |  | |  |  |  |
| *Controls* |  |  |  |  | |  |  |  |
|  | WLSMV (mice) | 21.90 | 19.08 | 16.90 | | [0.26; 1; 4.31] | [1.21 ; 11.14; 1429] | substantial |
|  | WLSMV (pairwise) | 21.36 | 18.93 | 11.37 | | [0.26 ; 1 ; 4.31] | [1.21 ; 11.14 ; 1429] | substantial |
|  | ML (mice) | 23.94 | 23.36 | 1.78 | | [0.25; 1; 4.51] | [0.83; 1.74; 23.64] | none |
|  | FIML | 26.04 | 24.87 | 3.20 | | [0.26; 1; 3.81] | [1; 3.30; 148.91] | none |
|  |  |  |  |  | |  |  |  |
| *Note.* LL hypothesis = log-likelihood of H1, LL complement = log-likelihood of the complement, gw ratio = GORICA weight ratio, 5% ; 50% ; 95% null = percentiles of the null distribution; 5% ; 50% ; 95% obs = percentiles of the observed GORICA weight distribution, support = verbal interpretation of the results. All penalty weights are .50. | | | | | | | | |

| **Table S7 \| Nested models with corresponding parameter restrictions.** | | | | | |
| --- | --- | --- | --- | --- | --- |
| *Paths included* | *χ^2^* | *df* | *RMSEA* | *TLI* | *SRMR* |
| H1 \| H2.1 \| H2.2 \| H3.1 \| H3.2 | 2597 | 266 | 0.069 | 0.468 | 0.095 |
| **H1** \| H2.1 \| H2.2 \| H3.1 \| H3.2 | 1977 | 255 | 0.049 | 0.467 | 0.069 |
| **H1** \| **H2.1** \| H2.2 \| H3.1 \| H3.2 | 1950 | 253 | 0.049 | 0.471 | 0.071 |
| **H1** \| **H2.1** \| **H2.2** \| H3.1 \| H3.2 | 1948 | 252 | 0.049 | 0.469 | 0.071 |
| **H1** \| **H2.1** \| **H2.2** \| **H3.1** \| H3.2 | 1949 | 251 | 0.049 | 0.466 | 0.071 |
| **H1** \| **H2.1** \| **H2.2** \| **H3.1** \| **H3.2** | 1959 | 254 | 0.049 | 0.470 | 0.073 |
| H1 \| **H2.1** \| **H2.2** \| **H3.1** \| **H3.2** | 1962 | 255 | 0.049 | 0.472 | 0.073 |
| H1 \| **H2.1** \| **H2.2** \| H3.1 \| **H3.2** | 2002 | 256 | 0.049 | 0.462 | 0.069 |
| H1 \| **H2.1** \| H2.2 \| **H3.1** \| **H3.2** | 1959 | 254 | 0.049 | 0.470 | 0.073 |
| H1 \| H2.1 \| **H2.2** \| **H3.1** \| **H3.2** | 2006 | 257 | 0.049 | 0.463 | 0.069 |
| H1 \| **H2.1** \| H2.2 \| H3.1 \| **H3.2** | 1977 | 255 | 0.049 | 0.467 | 0.069 |

1. **Imputation of missing values—assumption checks**

Since the mice imputation procedure assumes that data are missing at random (MAR), we checked whether we would be able to detect systematic predictors of missingness that would potentially bias the estimation of our focal Granger-causal relationship. Specifically, we checked whether material security at Wave 1, belief in God at Wave 1, or their interaction predict missing answer on belief in God at Wave 4. In other words, our worry was that if participants low on material security would drop their belief in God, they might be more likely to discontinue taking part in the survey (unlike materially secure individuals who might continue). Such a process might bias our inference, selecting out materially insecure disbelievers.

To test this possibility, we regressed the binary variable recoding missingness in belief in God in Wave 4 (missing/remained) on material security in Wave 1, belief in God in wave 1 (dummy-coded, no belief as reference), and their interaction. The logistic regression showed that material security indeed had negative effect on missingness for participants who reported no belief in God in Wave 1 (OR = 0.79; 95% CI = [0.65; 0.89]; p = .001). The effect of material security on drop-out was weaker for those reporting uncertain belief in God in Wave 1 (changing to OR = 0.93) compared to those who answered positively (OR = 0.84). Nevertheless, this suggests that material insecurity predicted dropping from the survey for both believers and non-believers. In combination with other variables in the dataset, this suggests presence of systematic missingness patterns which allow multiple imputation. Hence, we proceeded with imputation and the imputation details for each variable are reported in Table S8. As noted in the main text, the imputation procedure did not yield valid values for 3 participants. Based on our diagnostics of the issue, it appears that these participants lacked valid values in multiple variables that were used as imputation predictors, especially in Wave 4. This issue can be solved by making the imputation model sparser (e.g., removing Wave 4 predictors). On the other hand, this approach would risk losing information for the participants whose values were imputed successfully, making the imputation procedure less accurate as a result. Therefore, we decided to accept losing these three participants instead of reducing the imputation model.

**Table S8 | Summary of the imputation procedure.**

| Variable | Missing % | Imputation Method | Predictor |
| --- | --- | --- | --- |
| Gender | 0 |  | TRUE |
| PRACE | 1.2 |  | TRUE |
| ETHRACE | 3.5 | polyreg | TRUE |
| Age | 0 | pmm | TRUE |
| MS1 | 6.1 | polr | TRUE |
| BiG1 | 0 |  | TRUE |
| PEDUC1 | 0.1 |  | FALSE |
| PSPEDUC | 28.2 |  | FALSE |
| PEDUC3 | 31.5 |  | FALSE |
| ParRit | 0.1 | polr | TRUE |
| PST | 0.2 | polr | TRUE |
| RELTRAD | 0 | polyreg | TRUE |
| PSPEDUC3 | 0 |  | FALSE |
| ParCatholic | 0 |  | TRUE |
| ParProtestant | 0 |  | TRUE |
| BiG2 | 23.2 | polr | TRUE |
| H2 | 23.4 | polr | TRUE |
| CR2_1 | 23 |  | FALSE |
| CR2_2 | 44.7 |  | FALSE |
| PR2 | 23.5 | polr | TRUE |
| T2 | 23.8 | polr | TRUE |
| BiG3 | 25.1 | polr | TRUE |
| H3 | 25.2 | polr | TRUE |
| CR3_1 | 24.9 |  | FALSE |
| CR3_2 | 52.4 |  | FALSE |
| PR3 | 25.2 | polr | TRUE |
| Inc3 | 27.6 | pmm | TRUE |
| BiG4 | 36.6 | polr | TRUE |
| H4 | 36.6 | polr | TRUE |
| CR4_1 | 36.6 |  | FALSE |
| CR4_2 | 69.7 |  | FALSE |
| PR4 | 36.7 | polr | TRUE |
| Inc4 | 39 | pmm | TRUE |
| EDATT_W4 | 36.6 | polr | TRUE |
| FirstParEd | 0.1 |  | FALSE |
| SecondParEd | 28.2 |  | FALSE |
| ParEd | 0 |  | FALSE |
| ParEd_ord | 0.1 | polr | TRUE |
| Male | 0 | pmm | FALSE |
| AsianPR | 1.2 |  | FALSE |
| BlackPR | 1.2 |  | FALSE |
| LatinxPR | 1.2 |  | FALSE |
| NativePR | 1.2 |  | FALSE |
| OtherPR | 1.2 |  | FALSE |
| CR2 | 23 | polr | TRUE |
| CR3 | 24.9 | polr | TRUE |
| CR4 | 36.6 | polr | TRUE |
| BiG4NA | 0 |  | FALSE |

*Note*. pmm = predictive mean matching, polr = proportional odds model, polyreg = polytomous regression, no method listed = variable was not imputed. Variables not listed were used as neither the predictor nor imputation target.

1. **Deviations from pre-registration**

| **Table S9 \| Deviations from pre-registration.** | | | | | |
| --- | --- | --- | --- | --- | --- |
| # | **Details** | | **Original Wording** | **Deviation Description** | **Reader Impact** |
| **1** | Type | Hypotheses | H2.1: Better self-reported health at Wave 2 predicts participants’ lower self-reported belief in God at Wave 4.  H2.2: Higher trust toward others at Wave 2 predicts participants’ lower self-reported belief in God at Wave 4.  Mechanisms of religious systems mediating the effects of exogenous pressures:  H3.1: The negative effect of self-reported health at Wave 2 on self-reported belief in God at Wave 4 is mediated by the frequency of prayer at Wave 3.  H3.2: The negative effect of trust in others at Wave 2 on self-reported belief in God at Wave 4 is mediated by the frequency of religious service attendance at Wave 3. | Following reviewers’ comments, we concluded that neither the dataset or the DAG we set up allow us to make causal claims about Health and Trust variables. Therefore, we decided to focus solely on the main causal path (H1) for which the DAG was designed in the main text and moved the test of these hypotheses into the supplements where we still report them but caution readers that these results cannot afford causal inference. | The mechanisms through which material security may decrease belief in God later in life are tentative and need further research that allows causal claims. |
|  | Reason | Peer review |  |  |  |
|  | Timing | After results known |  |  |  |
| **2** | Type | Research Q(s) | 2) Does self-reported health quality cause disbelief?  3) Does perception of greater trust in others cause disbelief?  4) Does the frequency of personal prayer mediates the effect of health on disbelief?  5) Does the frequency of religious service attendance mediates the effect of trust on disbelief? | Same as above | Same as above |
|  | Reason | Peer review |  |  |  |
|  | Timing | After results known |  |  |  |
| **3** | Type | Analysis |  | Due to the above deviations, we do not report the full model we refer to in our preregistered code and the pdf in the registration’s repository. However, a version of this model is reported in the supplement. | Same as above |
|  | Reason | Peer review |  |  |  |
|  | Timing | After results known |  |  |  |

| **4** | Type | Analysis | Finally, as a robustness check, we will also corroborate these results by fitting several nested models that refit the full model with the paths negated by each theory fixed to 0. We will then select the best-fitting model based on RMSEA, SRMR, AIC, and BIC. | AIC/BIC can only be compared for models estimated using maximum likelihood. Therefore, we do not report AIC/BIC in this step and use SRMR, RMSEA and TLI instead. Given the deviations noted above, this part of the analysis has been moved completely to the supplement. | Same as above |
| --- | --- | --- | --- | --- | --- |
|  | Reason | Plan not possible |  |  |  |
|  | Timing | After data access |  |  |  |

| **Table S10 \| Unregistered steps.** | | | | | |
| --- | --- | --- | --- | --- | --- |
| # | Details | | Original Wording | Unregistered Step Description | Reader Impact |
| **1** | Type | Data Preparation | We will listwise exclude participants with missing data on focal variables for each hypothesis test. If these listwise deletions exceed 10% of the sample, we will use multivariate imputation techniques to impute the missing data. | Based on a reviewer’s comment, we now conduct a more thorough missing data treatment, including more detailed reporting on the imputation procedure and assessing missingness patterns. These steps were added post hoc and were not preregistered. | The results are now more robust due to a more precise treatment of missing data. |
|  | Timing | After results known |  |  |  |
| **2** | Type | Data Preparation |  | Before analysis, we exclude (1) non-Christian participants and (2) participants who do not report “Yes” in Belief in God in Wave 1. Data including (2) are however used in the imputation procedure. These steps were not registered but performed before analysis. | Our conclusions relate only to Christians and only to those who believed in God in Wave 1. We cannot conclude anything about Christians who would report believing in God later in life. |
|  | Timing | Before data access |  |  |  |
| **3** | Type | Analysis | Finally, as a robustness check, we will also corroborate these results by fitting several nested models that refit the full model with the paths negated by each theory fixed to 0. We will then select the best-fitting model based on RMSEA, SRMR, AIC, and BIC | The robustness checks have been extended to include different missing data treatments (listwise, pairwise, FIML, multiple imputation) and ordinal vs. continuous treatment of the response variables. | The results are now more transparent because the reader can assess how our analytical choices impacted the conclusions. |
|  | Timing | After results known |  |  |  |
| **4** | Type | Analysis |  | Based on a reviewer’s comment, we begin our analyses by checking what is the effect of Material Security on Belief in God in Wave 4 when (1) we consider only these two variables and (2) after we add controls. | The reader is now informed about the magnitude and the direction of the effect from H1 without additional controls and analytical steps. However, they should still use our main model to gain the most accurate estimate of the H1 effect. |
|  | Timing | After results known |  |  |  |
|  | **5** | Data coding  Before analysis |  | We collapsed the no degree and high school options since the no degree option had only very small representation in the overall sample (3.8%). | This step should have no effect on the main results, but allowed better model fit. |
|  | **6** | Analysis  After results known |  | In the model with controls, we added paths from parent ritual frequency in Wave 1 to belief in God in Waves 1-3. | This modification of our model was suggested by a reviewer, and we agree that it makes logical sense that parents’ ritual attendance during participants’ adolescence may exert continues influence on belief in God during all waves. |
|  | **7** | Analysis  After results known |  | In the model with controls, we let the correlations between the levels of categorical variables to be freely estimated (for religious tradition and education) and also allowed the model to estimate the correlation between College and Income in Wave 3. | These model modifications were motivated by the diagnostics of our model due to low TLI value.o |

1. **References:**

Hardie, J. H., Pearce, L. D., & Denton, M. L. (2016). The Dynamics and Correlates of Religious Service Attendance in Adolescence. *Youth & Society*, *48*(2), 151–175. https://doi.org/10.1177/0044118X13483777

Mikoski, C., & Olson, D. V. A. (2021). Does Religious Group Population Share Affect the Religiosity of the Next Generation? *Journal for the Scientific Study of Religion*, *60*(3), 611–627. https://doi.org/10.1111/jssr.12727

Perry, S. L., & Longest, K. C. (2019). Examining the Impact of Religious Initiation Rites on Religiosity and Disaffiliation over Time. *Journal for the Scientific Study of Religion*, *58*(4), 891–904. https://doi.org/10.1111/jssr.12632

Rhemtulla, M., Brosseau-Liard, P. É., & Savalei, V. (2012). When can categorical variables be treated as continuous? A comparison of robust continuous and categorical SEM estimation methods under suboptimal conditions. *Psychological Methods*, *17*(3), 354–373. https://doi.org/10.1037/a0029315

Schwadel, P. (2008). Poor Teenagers’ Religion. *Sociology of Religion*, *69*(2), 125–149.

Schwadel, P. (2016). Does Higher Education Cause Religious Decline?: A Longitudinal Analysis of the Within- and Between-Person Effects of Higher Education on Religiosity. *The Sociological Quarterly*, *57*(4), 759–786. https://doi.org/10.1111/tsq.12153

Smith, J. (2021). Transmission of Faith in Families: The Influence of Religious Ideology. *Sociology of Religion*, *82*(3), 332–356. https://doi.org/10.1093/socrel/sraa045
